# Artificial Intelligence Tools in Pre-Travel Health Consultations: A Scoping Review of Clinical Evidence, Implementation Gaps, and Emerging Opportunities

**DOI:** 10.3390/tropicalmed11070186

**Published:** 2026-07-06

**Authors:** Haider Saddam Qasim, Maree Donna Simpson

**Affiliations:** School of Medical Sciences and Dentistry, Charles Sturt University, Orange Campus, Leeds Parade, Orange, NSW 2800, Australia

**Keywords:** pre-travel health consultations, artificial intelligence, large language models, travel medicine, clinical decision support, patient safety, digital health

## Abstract

Background: Pre-travel health consultations require individualised risk assessment across itinerary, destination, traveller characteristics, vaccine and medication history, comorbidities, pregnancy and immune status, activities, and access to care. Artificial intelligence (AI), particularly large language models (LLMs), may support pre-consultation education, structured history collection, guideline retrieval, multilingual communication and post-consultation reinforcement, but unsafe use may introduce hallucinated, outdated or insufficiently personalised recommendations. Objectives: This scoping review maps the current evidence on AI tools relevant to pre-travel health consultations, characterises implementation gaps, identifies patient-safety risks and proposes a supervised implementation model for travel medicine clinics. Original contribution: Unlike previous reviews of clinical AI, patient-education LLMs or chatbots in chronic illness, this is the first scoping review focused specifically on AI in pre-travel consultations. It uniquely combines a five-tier evidence hierarchy that separates direct travel-medicine AI evidence from indirect clinical-AI safety and equity evidence, and provides a travel-medicine-specific clinical safety risk taxonomy and a supervised implementation framework anchored to authoritative travel-medicine guidance and current AI regulatory regimes. Methods: A scoping review was conducted following PRISMA-ScR reporting, using a Population–Concept–Context eligibility framework and a targeted retrieval in May 2026 covering January 2017 to May 2026. Sources were screened and charted by a single reviewer using a structured eligibility checklist. Quality and applicability were appraised conceptually using MMAT, AMSTAR 2 and JBI text-and-opinion criteria, with GRADE-informed certainty. Results: Of 70 records identified, 11 were included: four direct pre-travel AI sources, one adjacent travel-related decision-support study, four guideline and context sources and two clinical LLM safety sources. The only patient-level implementation involved 26 travellers using a GPT-4 Travel Clinic Assistant in Singapore, where physicians and travellers reported acceptability and workflow benefit but objective effectiveness outcomes were not measured. Broader clinical LLM evidence indicates heterogeneous evaluation methods, vulnerability to hallucinated guidelines, and accuracy that varies widely across model versions and specialties. Conclusions: Current evidence supports supervised AI augmentation of pre-travel consultations but does not support autonomous AI-led vaccine selection, malaria prophylaxis, contraindication screening or individualised travel-risk clearance. Near-term deployment should be restricted to clinician-supervised education, structured intake, source-grounded guideline retrieval, after-visit reinforcement and escalation-triggered workflow support. Priority research includes travel-medicine-specific hallucination audits; equity testing in visiting-friends-and-relatives, migrant, older-adult, First Nations Australian, and Pacific Islander travellers; and prospective trials reported under CONSORT-AI, SPIRIT-AI and TRIPOD + AI.

## 1. Introduction

Pre-travel consultation is a preventive clinical encounter that synthesises traveller characteristics, destination-specific risks, itinerary, trip duration, purpose of travel, activities, timing, medical history, medications, immunisation history, and special risk states such as pregnancy or immunocompromised status [1]. Core interventions include immunisation, malaria chemoprophylaxis, risk communication, food and water safety advice, vector avoidance, injury prevention, respiratory infection precautions, blood-borne infection prevention, and sexual-health counselling [1].

The International Society of Travel Medicine emphasises that pre-travel care is broader than vaccination alone and should account for destination, activities, age, health status, chronic disease, past vaccines, medication requirements, and timely advice approximately six weeks before travel where possible [2]. WHO travel-health guidance similarly highlights malaria geography, chemoprophylaxis, mosquito personal protection, and treatment considerations as central components of international travel health [3].

Pre-travel consultation uptake remains uneven globally. Surveys in primary care and migrant communities identify perceived low risk, awareness gaps, time and cost barriers, and language barriers as the principal obstacles to pre-travel care, with visiting-friends-and-relatives (VFR) travellers and migrant populations particularly underserved [4,5,6,7]. Primary care physicians, who manage many travellers, often report low travel-medicine knowledge and limited consultation time [7]. These structural gaps create the context in which AI may plausibly assist, provided it does not replicate or worsen access inequities.

AI is rapidly transforming clinical practice across diagnostic imaging, decision support, health monitoring, and patient engagement [8]. Travel medicine appears attractive for AI augmentation because it is guideline-intensive, time-sensitive, multilingual, and highly dependent on structured information [9,10]. The GeoSentinel surveillance network provides a globally distributed data substrate that could plausibly support AI-enabled outbreak detection and destination-specific risk modelling [11,12]. However, the same features that make travel medicine attractive for AI also make unsafe automation problematic. A generic answer that omits splenectomy, live-vaccine contraindications, pregnancy, immunosuppression, HIV status, transplant history, or region-specific malaria variation could create clinically meaningful harm [1,3,13,14].

Although clinical AI has been reviewed in adjacent areas—large language models in patient education [15], chatbots in chronic illness management [16], healthcare chatbots more broadly [17] and preventive-care chatbot outreach [18]—none of these reviews has the pre-travel consultation as its primary subject, none maps the travel-medicine-specific evidence base against authoritative travel-medicine guidance [7,8,9,19], and none situates the evidence within current AI regulatory regimes [20,21,22,23] or proposes a supervised implementation model for travel clinics. Existing travel-medicine AI work is confined to four direct sources—a ChatGPT-4 pre-travel advice evaluation [23], a single 26-traveller GPT-4 implementation in Singapore [24], a design decalogue with a pre-alpha prototype [25] and editorial pieces framing supervised augmentation [9,10]—leaving a clear gap between rapidly emerging AI tools and the structured, safety-oriented synthesis travel medicine practice needs. This review therefore asks what AI tools have been evaluated in or near pre-travel health consultations, what the current evidence permits clinicians to conclude, what patient-safety and implementation gaps remain, and how travel clinics might deploy AI without replacing specialist clinical judgement. By doing so, it offers the first scoping review focused specifically on pre-travel AI, an explicit evidence-tier structure that separates direct travel-medicine AI evidence from indirect supporting evidence, a travel-medicine-specific safety taxonomy, and a supervised implementation framework that can be tested in subsequent prospective work.

## 2. Methods

### 2.1. Review Design

This review uses a scoping review methodology, which is appropriate when the goal is to map the extent, range, and nature of evidence in an emerging and heterogeneous field rather than estimate intervention effects or conduct meta-analysis [1,26,27,28]. The review follows the Arksey and O’Malley framework as advanced by Levac and colleagues and operationalised through JBI guidance, with reporting aligned to PRISMA-ScR [1,26,27,28]. A completed PRISMA-ScR checklist with manuscript locations is provided as Supplement S1. The review was not prospectively registered.

### 2.2. Population, Concept, Context (PCC) Framework

The eligibility framework follows the JBI PCC structure for scoping reviews [29]. Table 1 defines the framework operationalised in this review.

### 2.3. Eligibility Criteria

Sources were eligible if they met one or more of the following criteria, mapped to the PCC framework:

- AI or LLM tools used for pre-travel health advice, pre-travel counselling, travel clinic education, or travel-medicine workflow support [1,2,3,4,5,6,10].
- AI chatbot design frameworks for travel medicine [25].
- Travel-related clinical decision-support systems relevant to travel medicine workflows [30].
- Authoritative guidelines defining the standard of care for pre-travel consultation [7,8,9,19].
- General clinical LLM safety, hallucination, and evaluation literature directly relevant to clinical decision support [11,12,13,14,20,31,32].
- Adjacent preventive-counselling literature where it informed implementation design, equity, or evaluation standards [15,16,33,34,35,36,37,38].

Sources were excluded if they were unrelated to AI, unrelated to travel medicine or clinical decision support, non-healthcare AI papers, unrelated travel behaviour studies without AI relevance, or purely technical AI papers without clinical or implementation relevance.

### 2.4. Search Strategy

This review used a transparent, targeted scoping retrieval rather than a fully reproducible multi-database systematic search. Retrieval was conducted in May 2026 over the date range January 2017 to May 2026 using the following components, all documented in Supplement S2:

- One direct PubMed/MEDLINE search string combining travel-medicine and AI/LLM concepts.
- Academic and web-indexed search tools applied to the same concept set, including searches restricted to authoritative travel-medicine and clinical AI domains.
- Citation chasing on the four direct travel-medicine AI sources [1,2,3,4,5,6] and on the FeverTravelApp study [30].
- Targeted retrieval from authoritative guideline and regulator websites (CDC, WHO, ISTM, FDA, TGA, European Commission/EU AI Act portal) [7,8,9,19,20,21,22,23,24,25,39].
- Hand retrieval from *Journal of Travel Medicine*, *Travel Medicine and Infectious Disease*, *BMC Digital Health*, and *Communications Medicine* for AI-relevant titles within the search window.

The PubMed/MEDLINE string used was

(“travel medicine”[Title/Abstract] OR “pre-travel”[Title/Abstract] OR “pretravel”[Title/Abstract] OR “travel health”[Title/Abstract] OR “traveller health”[Title/Abstract]) AND (“artificial intelligence”[Title/Abstract] OR “large language model”[Title/Abstract] OR “ChatGPT”[Title/Abstract] OR chatbot*[Title/Abstract] OR “clinical decision support”[Title/Abstract] OR “machine learning”[Title/Abstract])

Complementary search strings for Embase, CINAHL, Cochrane CENTRAL, IEEE Xplore, ACM Digital Library, ClinicalTrials.gov and the WHO ICTRP are documented in Supplement S2 as recommended reproducibility strategies. These additional databases were planned but not executed for this scoping retrieval, and this restriction is recorded transparently as a significant methodological limitation. It increases the risk that relevant records—particularly in computer-science venues indexed by IEEE Xplore and the ACM Digital Library, nursing and allied-health literature indexed by CINAHL, and ongoing or unpublished trials indexed by ClinicalTrials.gov and the WHO ICTRP—were not retrieved. Readers should therefore interpret the included evidence base as a lower bound on what is available, and the synthesis as a field map rather than a comprehensive systematic review. A full multi-database run is identified as a high-priority requirement for any subsequent confirmatory review [1,26,27,28].

### 2.5. Study Selection and Data Charting

Screening and data charting were conducted by a single reviewer (the manuscript author) using a structured eligibility checklist. Because screening was conducted by a single reviewer, Cohen’s kappa for inter-rater agreement was not calculable. Mitigation strategies included a written eligibility checklist (Supplement S2), explicit decision rules for ambiguous cases, and second-pass review of borderline records by the same reviewer at least 24 h after the first pass. Charted fields included article type, country and setting, AI/tool type, sample size, comparator or reference standard, outcomes, findings, safety concerns, implementation implications, and relevance to specific pre-travel consultation tasks. Single-reviewer screening is a known threat to validity in scoping reviews and is acknowledged as a limitation [1,26,27,28]. The full charting table for the 11 included sources is provided as Supplement S4.

### 2.6. Quality Appraisal and Certainty Assessment

Scoping reviews do not require formal risk-of-bias assessment, but an indicative quality and applicability appraisal was performed to inform interpretation and certainty rather than inclusion [26,29]. No source was excluded on the basis of quality appraisal. The Mixed Methods Appraisal Tool (MMAT) was used conceptually for empirical mixed or quantitative implementation evidence because it covers qualitative, randomised, non-randomised, quantitative descriptive, and mixed-methods studies [40]. AMSTAR 2 principles were used to interpret systematic reviews [41]. GRADE categories were used cautiously as “GRADE-informed certainty” because GRADE reflects confidence that observed evidence approximates the true effect and considers risk of bias, inconsistency, indirectness, imprecision, and publication bias [42]. Editorials and guideline texts were appraised using JBI text-and-opinion guidance [29].

### 2.7. Study Selection Flow

**Figure 1:**
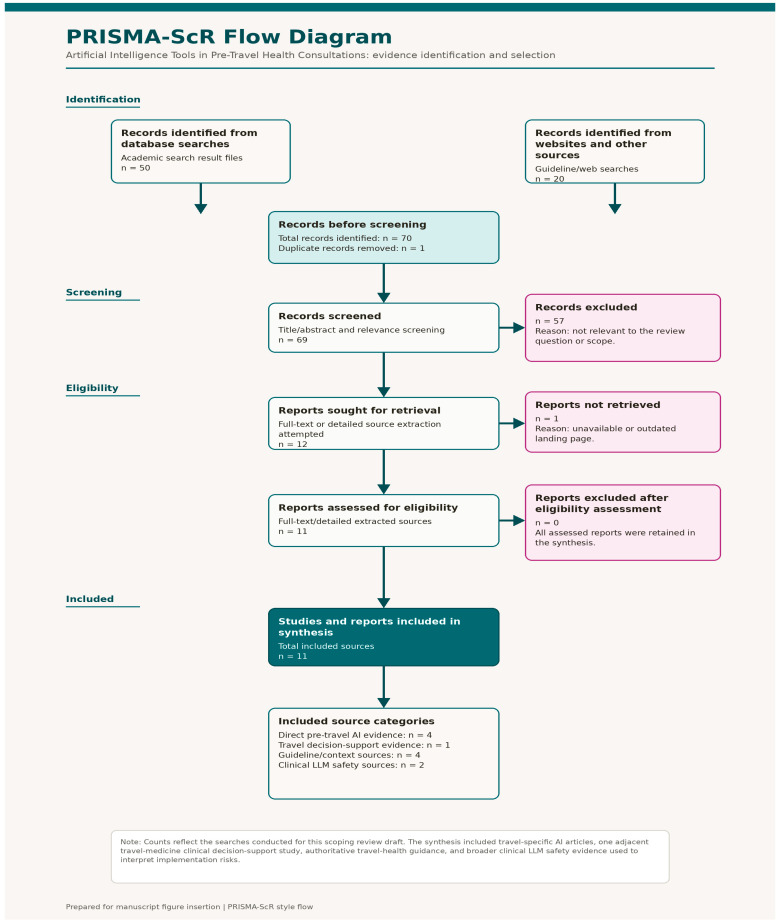
PRISMA-ScR style flow diagram summarising identification, screening, eligibility assessment and included source categories for the scoping review of AI tools in pre-travel health consultations [26]. The 57 records excluded at title-and-abstract screening were post hoc classified into six mutually exclusive categories reconstructed from the screening notes: (i) AI in non-travel clinical domains (~22 records); (ii) AI in non-clinical or general-purpose contexts (~10); (iii) travel behaviour or epidemiology without AI relevance (~12); (iv) general AI methodology without clinical implementation content (~7); (v) duplicate or near-duplicate coverage of an already-included source (~4); and (vi) editorials, opinion or letters not addressing AI in or near pre-travel consultation (~2). One record was sought but not retrieved within the search window and is recorded in Supplement S3. No reports were excluded after full-text assessment.

The search identified 70 records across PubMed/MEDLINE, academic and web-indexed sources, citation chasing, and authoritative guideline and regulator retrieval. After removal of one duplicate, 69 records were screened; 57 were excluded at title-and-abstract screening, 12 reports were sought for retrieval, one report could not be retrieved within the search window and 11 reports were assessed in full text and included in the synthesis. Included evidence comprised four direct pre-travel AI sources [1,2,3,4,5,6], one travel-related decision-support source [30], four guideline and context sources [7,8,9,19], and two clinical LLM safety sources [13,43]. To increase methodological transparency, the 57 excluded records were post hoc classified by the single reviewer into six mutually exclusive exclusion categories, reconstructed from the screening notes and tabulated in full in Supplement S3: (i) AI applied in non-travel clinical domains without transferable travel-medicine implications (approximately 22 records—for example, imaging, oncology, radiology, mental health or general primary care AI); (ii) AI in non-clinical or general-purpose contexts with no travel-medicine relevance (approximately 10 records—for example, AI in education, hospitality, tourism marketing or generic chatbot design); (iii) travel behaviour, travel epidemiology or post-travel illness studies without an AI or decision-support component (approximately 12 records); (iv) general AI methodology, technical machine-learning or large-language-model papers without a clinical implementation or evaluation component (approximately 7 records); (v) duplicate or near-duplicate coverage of an already-included source, including preprints subsequently superseded by published versions (approximately 4 records); and (vi) editorials, opinion or letters not addressing AI in or near a pre-travel consultation context (approximately 2 records). One additional record proceeded to retrieval but could not be obtained within the search window and is recorded in Supplement S3. No reports were excluded after full-text assessment. The PRISMA-ScR flow diagram summarising the identification, screening, eligibility and inclusion steps is presented in Figure 1.

## 3. Results

### 3.1. Confidence in the Evidence Base

A central finding of this review is the thinness of direct evidence. Only four sources directly address AI in travel medicine, and only one of these is a patient-level implementation study, in 26 travellers in a single Singapore tertiary travel clinic [24]. Four levels of evidence informed the synthesis with descending confidence in direct applicability:

Sources were assigned to evidence tiers using four pre-specified, mutually exclusive criteria applied in sequence to each included record: (a) subject of evaluation—whether the AI tool was evaluated, prototyped or implemented for the pre-travel consultation itself, an adjacent travel-related clinical workflow, or a non-travel clinical task; (b) data source—whether outcomes were observed in actual travellers, in simulated patients or expert raters, or were not empirical; (c) document type—empirical study, framework or design paper, expert opinion or editorial, authoritative guideline, or regulatory text; and (d) direction of transfer—whether findings transfer to pre-travel decision support directly, by analogy from adjacent clinical AI safety or accuracy evidence, or only as a reference standard or contextual constraint. Tier 1 required positive answers on all four criteria for the pre-travel domain; Tier 2 required (a) to have adjacent travel-related evidence rather than pre-travel; Tier 3 covered general clinical-AI safety and accuracy studies meeting (b) and (c) but not (a); Tier 4 covered guideline and regulatory texts; and Tier 5 covered adjacent implementation, equity and patient-engagement evidence. Tier assignments were applied by the single reviewer and are listed source-by-source in Table 2 and Supplement S4 so that readers can reproduce or contest each judgement; the criteria are not validated as a generic evidence-classification instrument and are offered as a transparent organising device for this review.

1. **Direct travel-medicine AI evidence (four sources):** The ChatGPT pre-travel advice evaluation [23], the Singapore Travel Clinic Assistant implementation [24], the Baglivo decalogue prototype [25], and the Flaherty editorials on supervised generative AI integration and the natural history of AI in travel medicine [9,10]. Heidema et al. is treated as adjacent rather than direct because it concerns AI-supported outbreak surveillance rather than the pre-travel consultation itself [12].
2. **Adjacent clinical AI evidence (eight sources):** Clinical LLM evaluation methods [43]; multi-model hallucination and clinical guideline omission/hallucination assurance analyses [13,34]; clinical documentation hallucination framework [14]; ChatGPT meta-analysis [35]; ChatGPT care-seeking accuracy across model versions [36]; ChatGPT FAQ literature review [44]; and travel-related clinical decision support [30].
3. **Guideline, regulatory, and methodology evidence (twelve sources):** CDC Yellow Book pre-travel guidance and VFR chapter [1,19]; ISTM pre-travel advice [2]; WHO malaria travel guidance [3]; PRISMA-ScR, scoping methodology, MMAT, AMSTAR 2, GRADE [1,17,18,26,27,28,33]; WHO AI ethics and digital health strategy [39,45]; FDA, TGA, and EU AI Act materials [20,21,22,23]; and AI reporting standards CONSORT-AI, SPIRIT-AI, and TRIPOD + AI [29,30,42,43].
4. **Adjacent implementation, equity, and patient-engagement evidence (ten sources):** Clinician adoption of AI [46]; AI in medical education [47]; AI in healthcare overview [8]; VFR uptake studies [4,5]; pre-travel consultation in primary care [6,7]; LLM patient education and chronic-illness chatbot reviews [15,16]; AHRQ healthcare chatbot review [17]; preventive-care chatbot outreach [18]; digital divide and equity [40,41,45]; and retrieval-augmented generation [49].

What is not known is therefore as important as what is known. There are no prospective travel-medicine AI trials reporting clinical safety or vaccine uptake outcomes, no published efficacy comparisons against standard travel-medicine consultation, no equity-focused trials in VFR, migrant, First Nations Australian, or Pacific Islander travellers, and no published commercial or open travel-medicine AI tools with regulatory approval as Software as a Medical Device for travel-medicine indications.

### 3.2. Evidence Base Overview

Four sources directly address AI in travel medicine: a ChatGPT pre-travel advice evaluation [23], a GPT-4 Travel Clinic Assistant implementation in Singapore [24], a proposed travel-health chatbot design decalogue [25], and editorials on integrating generative AI safely into travel medicine [9,10]. A separate travel-related clinical decision-support study examined a tablet-based algorithm for returned febrile travellers [30], and an additional editorial-tier source describes AI-supported outbreak detection using GeoSentinel data as a near-future adjacent application [12].

Only one direct travel-medicine AI implementation report included actual travellers. In that report, 26 travellers used a GPT-4 Travel Clinic Assistant in a Singapore tertiary travel clinic, and physician and traveller feedback suggested acceptability, improved focus of consultation, and perceived knowledge benefit, but there was no objective measurement of knowledge gain, clinical appropriateness, vaccine uptake, prophylaxis adherence, adverse events, or downstream travel illness [24].

The broader evidence base supports caution. A systematic review of 761 clinical LLM evaluations found heterogeneous methods and limited standardisation of evaluation rubrics [43]. A multi-model assurance analysis showed LLMs repeated or elaborated planted false clinical details in 50 to 82 percent of simulated clinical prompts [13]. A clinical-documentation hallucination framework demonstrated that hallucinations are more likely than omissions to be classified as major errors, especially in the clinical plan section [14]. A diagnostic LLM analysis found measurable prevalence of clinical guideline omission and hallucination in LLM outputs, including fabricated protocols mimicking authoritative sources [34]. A literature review of ChatGPT in clinical FAQs, recommendations, and symptom interpretation found accuracy ranging from 20 to 95 percent across nine studies [44]. A meta-analysis of ChatGPT in medical and dental research described an 18 to 100 percent accuracy range across specialties [35]. An evaluation of ChatGPT model versions across 22 instances and multiple urgency levels found average accuracy near 70 percent, overtriage, and increasing variability with newer models [36].

### 3.3. Evidence Synthesis Table

Table 3 summarises the included sources, their methods, sample sizes, AI/tool types, the consultation tasks they map to in the CDC Yellow Book taxonomy [1], main findings, key safety concerns, and a GRADE-informed certainty rating with explicit reasoning [42].

### 3.4. Quality and Applicability Appraisal

Quality and applicability were appraised using MMAT-informed [40], AMSTAR 2-informed [41], JBI text-and-opinion-informed [29], and GRADE-informed [42] approaches. No source was excluded on the basis of quality appraisal; appraisal informed interpretation and certainty only. The results of this appraisal are summarised in Table 4.

### 3.5. What the Evidence Allows and Does Not Allow

The evidence allows a cautious conclusion that AI can support pre-consultation education, structured question preparation, basic travel-health information delivery, and clinician-supervised workflow support [1,2,3,4,5,15,33]. It also supports the conclusion that current general-purpose chatbots are not sufficiently validated for unsupervised individualised travel medicine [13,14,23,35,36,44].

The evidence does not allow claims that AI improves vaccine uptake, reduces travel-associated illness, improves malaria prophylaxis adherence, safely screens contraindications, reduces adverse events, or can replace a travel medicine clinician [1,2,3,4,5,10]. Those outcomes have not been tested in adequately powered prospective studies meeting CONSORT-AI, SPIRIT-AI, or TRIPOD + AI standards [29,30,42,43].

### 3.6. Clinical Safety Risk Taxonomy

Drawing on the included LLM safety, hallucination, and accuracy literature [11,12,13,14,20,31,32], travel-medicine guidance [7,8,9,19,48], and the empirical travel-medicine implementations [23,24,30], the principal AI failure modes for pre-travel consultations are summarised in Table 5.

**Why hallucination matters for travel medicine.** Hallucination—the generation of plausible-sounding but unsupported or false outputs—is the central safety concern raised by every LLM-evaluation source included in this review [11,12,13,14,20,31,32] and by the only published direct travel-medicine evaluation [23]. Reported hallucination rates in clinical LLM testing have ranged from approximately 50% to over 80% of complex clinical queries, depending on task design and model version [13]. In a pre-travel consultation the consequences are not abstract: a single fabricated guideline citation, missed contraindication, or out-of-date outbreak claim can place a traveller in a yellow-fever- or malaria-endemic setting without appropriate protection.

**Mechanisms of hallucination relevant to clinical practice.** Four mechanisms recur across the included LLM literature and explain why pre-travel use is high-risk. First, training-data drift: General-purpose models are trained on data with a fixed cut-off and have no knowledge of new outbreaks, vaccine schedule updates, or guideline revisions issued after that date [14,43]. Second, retrieval-grounding failure: Even when a model is connected to an external source, it may fail to retrieve the correct guideline section, or it may paraphrase the source in a way that subtly alters the clinical meaning [14,34,49]. Third, false-premise propagation: If the traveller’s input contains an incorrect assumption (for example, that a destination is malaria-free), the model often elaborates confidently on that false premise rather than challenging it [13]. Fourth, plausibility bias: Large language models are optimised to produce fluent, well-formatted text, which makes hallucinated content read like authoritative clinical advice and reduces the chance that an untrained reader will detect the error [14,35,43].

**Types of hallucination observed in pre-travel use.** Five hallucination types are clinically relevant. (i) Fabricated guideline citations—The model invents a CDC, WHO, or ISTM reference that does not exist [34]. (ii) False destination or outbreak claims—The model declares a country malaria- or yellow-fever-free when current CDC Yellow Book or WHO data show ongoing transmission [3,11]. (iii) False reassurance for high-risk travellers—The model gives routine advice to an immunosuppressed, splenectomised, pregnant, or transplant traveller without flagging the need for specialist review [23,30]. (iv) Drug-interaction omission—The model fails to surface a relevant interaction (for example, between mefloquine and a chronic medication) because the interaction was not present in its retrieval window [13,14]. (v) Outdated outbreak status—The model continues to cite outbreak conditions that have either resolved or escalated since its training cut-off [11,12].

**Mitigations the implementation model relies on.** The supervised model in Section 3.7 and Table 6 is built around four hallucination mitigations grounded in the included literature: (1) retrieval-augmented generation, in which the model is constrained to answer only from a curated set of authoritative sources—the CDC Yellow Book [1], WHO and ECDC outbreak data [2,3], ISTM and CDC clinician guidance [1], and the destination-specific advisories cited at [11,49]; (2) source-link enforcement, so that every clinical claim displayed to the traveller or clinician is rendered with the underlying citation visible, allowing rapid verification; (3) date-stamped destination data and a model-version log, so that out-of-date outbreak content can be detected during the monthly audit defined in the quality-assurance row of Table 6; (4) hard-stop clinician review for any output touching a high-risk traveller category, a live vaccine, prescribing, or a flagged escalation trigger—that is, every row of Table 6 in which the failure-mode column is engaged. These mitigations do not eliminate hallucination, but they convert it from a silent risk to one the clinician can detect and contain.

**Figure 2:**
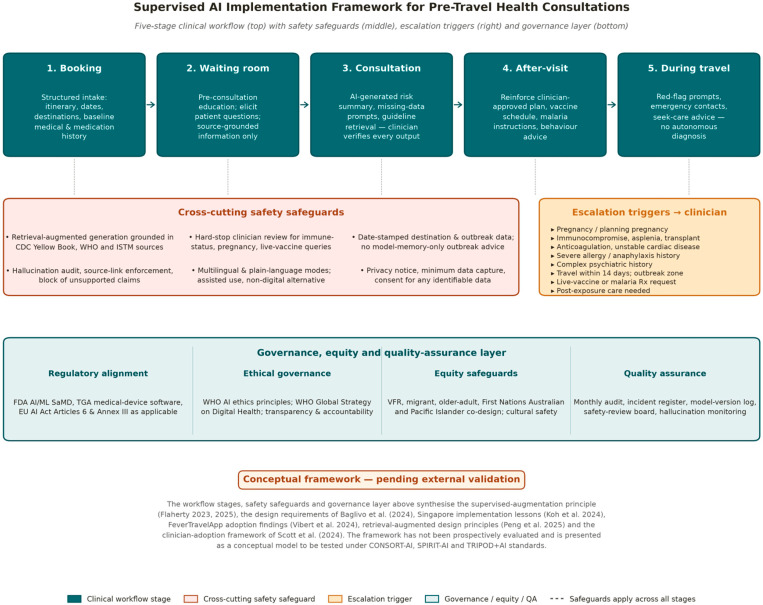
Schematic of the proposed supervised AI implementation framework for pre-travel health consultations. The five-stage clinical workflow (booking, waiting room, consultation, after-visit, during travel) is shown across the top. Cross-cutting safety safeguards (retrieval-augmented generation grounded in CDC, WHO and ISTM sources; hard-stop clinician review of immune-status, pregnancy and live-vaccine queries; date-stamped destination and outbreak data; hallucination auditing and source-link enforcement; multilingual and assisted-use modes; privacy and minimum-data-capture controls) apply to every stage. Escalation triggers route high-risk travellers to clinician review. The governance, equity and quality-assurance layer at the foot of the figure aligns the framework with FDA, TGA and EU AI Act requirements [20,21,22,33]; WHO AI ethics and digital health strategy [39,45]; equity co-design with visiting-friends-and-relatives, migrant, older-adult, First Nations Australian, and Pacific Islander travellers [4,5,19,32,38,48]; and monthly audit and hallucination monitoring [13,14,34,43,46]. The framework is a literature-informed conceptual model derived from Flaherty editorials [9,10], Baglivo et al. [25], Koh et al. [24], Vibert et al. [30], Peng et al. [49] and Scott et al. [46]. It has not been prospectively evaluated. ***Caveat.*** The implementation model presented in Table 6 and Figure 2 is a literature-informed synthesis, not a validated framework. None of its workflow stages, safety safeguards, escalation triggers or governance components has been evaluated in a prospective clinical study of AI in pre-travel consultations. The model should be treated as a conceptual scaffold for design, pilot, and trial work, to be tested and refined through stakeholder co-design and prospective evaluation under CONSORT-AI, SPIRIT-AI and TRIPOD + AI standards [20,31,33,37] before any clinical reliance is placed on it.

Section 3.7, Section 3.8, Section 3.10 and Section 3.12 present synthesis outputs derived from the included evidence rather than primary descriptive findings: a supervised implementation model (Table 6, Figure 2), a regulatory-landscape mapping (Table 7), exclusion criteria for AI as the primary interaction (Table 8) and a research priority matrix (Table 9). In line with Reviewer 2′s suggestion, these sections are interpretive extensions of the findings and are revisited in the Discussion (Section 4.9, Section 4.10, Section 4.11, Section 4.12) so that the reader can read them either as structured results or as discussion-tier syntheses. Their inclusion in Section 3 retains the tables’ and figures’ adjacency to the evidence they synthesise; their cross-references in Section 4 frame them explicitly as interpretive outputs.

### 3.7. Implementation Model for Travel Clinics (Interpretive Synthesis; See Also Section 4.9)

The implementation model in Table 6 synthesises the supervised-augmentation principle from the Flaherty editorials [9,10], the design requirements from Baglivo et al. [25], the Singapore implementation lessons [24], the FeverTravelApp adoption findings [30], retrieval-augmented design principles [49], and the clinician-adoption framework articulated by Scott and colleagues [46].

### 3.8. When Not to Use AI as the Primary Interaction (Interpretive Synthesis; See Also Section 4.11)

Table 8 summarises traveller characteristics where AI should not be the primary interaction. In these scenarios, AI may collect structured background information, but it should immediately escalate to clinician review [9,46].

### 3.9. Cost-Effectiveness and Implementation Feasibility

No included source reports cost-effectiveness data for AI in pre-travel consultations. Implementation feasibility considerations include licensing or hosting costs for clinical-grade LLM platforms, integration costs with electronic health records, ongoing prompt and retrieval-source maintenance, audit and governance staffing, multilingual content review, and equity-focused assistance for travellers with low digital literacy [24,25,46]. The chronic-illness chatbot review noted insufficient technical documentation in primary studies, which obstructs cost-effectiveness modelling and benchmarking [16]. The preventive-care chatbot evidence shows context-dependent efficacy and underperformance against telephone outreach overall, suggesting that travel-medicine AI value will likely be selective rather than uniform [18]. Future trials should report cost per consultation supported, cost per hallucination averted, equity-adjusted cost-effectiveness, and break-even thresholds for clinic deployment [29,30,42,43].

### 3.10. Regulatory Landscape (Interpretive Synthesis; See Also Section 4.10)

AI tools used in pre-travel consultations may fall within multiple medical-device and AI regulatory frameworks depending on intended use, claims, and risk class. Table 7 summarises the most directly relevant regimes.

### 3.11. Digital Equity Considerations

The Singapore implementation identified difficulty among some older adults because chatbot use required digital literacy [24]. This matters because travellers with chronic illness, older travellers, immunocompromised travellers, and people visiting friends and relatives may be both higher risk and more likely to face barriers to digital health tools [4,5,19].

VFR and migrant travellers are well-documented as high-risk and underserved. Qualitative work among VFR migrants identifies cultural perception of risk, financial barriers, language barriers, and trust as key obstacles to pre-travel care, and interventions that ignore these structural factors are unlikely to close the gap [4]. West African VFR travellers in the United States similarly under-utilise pre-travel care because of awareness, time, and cost barriers [5]. The CDC Yellow Book VFR chapter highlights that VFR travellers are disproportionately affected by malaria, hepatitis A, typhoid, and tuberculosis, and emphasises tailored counselling, language access, and family-inclusive education [19].

In Australia and the Pacific, First Nations Australians and Pacific Islander travellers face additional structural barriers, including geographic remoteness, primary care access gaps, and culturally specific information needs that are not addressed by generic English-language travel-medicine content; these populations are also more likely to travel for VFR purposes within and beyond the region. Direct empirical evidence on AI tools in these populations is absent in the included sources, which is itself a significant equity gap. Any travel-medicine AI deployment in Australia should therefore be co-designed with Aboriginal and Torres Strait Islander Community Controlled Health Services and Pacific community organisations rather than rolled out generically, and should be evaluated for cultural safety as well as accuracy [2,39,40,41,44,45,46].

Population-level data show persistent disparities in digital health care use. Impoverished, female, Black, and internet-poor populations experience reduced telehealth completion [32], county-level social vulnerability shapes 2022 digital health care use patterns [38], and the broader principle of “intervention-generated inequities” warns that new digital tools may inadvertently widen disparities if not deliberately designed for equity [48]. Migrant and VFR travellers in particular face multifaceted socioeconomic, financial, language, and systems-level barriers to pre-travel care [4,5,19].

Equity safeguards should include assisted kiosk use, clinician-reviewed non-digital alternatives, multilingual support [49], plain-language output, accessibility testing, culturally appropriate examples, and cost-free access. Travel-medicine AI training programmes should incorporate AI literacy and implementation science to support equitable deployment [47]. If AI is used to improve clinic efficiency but excludes high-risk travellers, it may worsen rather than reduce pre-travel health inequities [40,41,45].

### 3.12. Research Agenda and Priority Matrix (Interpretive Synthesis; See Also Section 4.12)

Future research priorities are summarised in Table 9. Trial-stage and prediction-model studies should follow CONSORT-AI [33], SPIRIT-AI [20,37], and TRIPOD + AI [31] reporting standards, ensuring that algorithm version, input data acquisition, human–AI interaction, fairness, subgroup performance, and post-deployment monitoring are reported consistently. A Minimum Reporting Standards checklist for travel-medicine AI studies is provided in Supplement S5, and a commercial and openly accessible tool inventory template, with current evidence limitations, is also in Supplement S5.

## 4. Discussion

This review supports a narrow but clinically important conclusion: AI can be useful in pre-travel health consultations as a supervised augmentation layer, but the evidence does not support autonomous AI decision-making [1,2,3,4,5,12,13,31,32,34]. The best-supported roles are education, question preparation, structured intake, guideline-linked summarisation, translation, literacy tailoring, and clinician-approved reminders [9,15,17,18,25,49].

### 4.1. Comparison with Prior Reviews and How This Review Extends Them

To the knowledge of the present review, no prior peer-reviewed scoping review specifically addresses AI tools in pre-travel health consultations as its primary subject. The Aydin et al. scoping review of LLMs in patient education spans general medicine and identifies recurring themes of patient education material generation, medical information interpretation, lifestyle recommendations, medication-use support, perioperative instructions, and doctor–patient interaction, while flagging readability, accuracy, and bias as cross-cutting challenges [15]. The Kurniawan et al. systematic review of chatbots for chronic illness reports promising acceptability but limited efficacy evidence and incomplete technical documentation [16]. The AHRQ review of healthcare chatbots similarly characterises the broader chatbot evidence as nascent [17]. The preventive-care chatbot retrospective analysis demonstrates that chatbot efficacy is selective and context-dependent, underperforming phone outreach overall while outperforming for diabetes care in 2023 [18].

The present review extends Baglivo et al. [25], who proposed a ten-item design decalogue for travel-health chatbots with a pre-alpha custom GPT prototype but did not synthesise empirical AI evidence in travel medicine, by mapping the empirical and adjacent evidence base, locating it within authoritative travel-medicine guidance and clinical AI safety standards, and embedding it in a supervised implementation model with explicit governance and equity safeguards. It extends the Flaherty editorials [9,10] by translating their supervised-augmentation principle into a structured workflow model and a clinical safety risk taxonomy. It extends Heidema et al. [12] by anchoring AI-enabled outbreak surveillance to the pre-travel consultation rather than treating it as a parallel activity. It extends generic clinical LLM safety work [11,12,13,14] by identifying which failure modes carry travel-medicine-specific consequences, particularly for live-vaccine eligibility, malaria prophylaxis, and outbreak-zone advice.

### 4.2. Why the Evidence Base Remains Thin

The thinness of the evidence base should shape, not weaken, the contribution of this review. A field-mapping review is valuable because it prevents premature clinical adoption from outrunning evidence. It also clarifies that travel medicine needs its own AI evaluation standards, rather than importing generic patient-education benchmarks from other specialties [15,16,43].

The main reason for caution is not that AI performs poorly on all travel-health questions. The ChatGPT evaluation suggests that general questions about food and water safety, vector avoidance, traveller’s diarrhoea, vaccine timing, and malaria concepts can be answered readably and often accurately [23]. The problem is that travel medicine becomes high-risk precisely when advice must be individualised to immune status, pregnancy, prior vaccines, comorbidities, medication interactions, destination micro-geography, and outbreak timing [1,3]. LLMs are particularly vulnerable to adversarial false-premise prompts and to hallucinated authoritative guidance in clinical decision support [12,13,14], and ChatGPT-class models show wide accuracy ranges across specialties and care-seeking tasks [35,36,44].

***Missing data types.*** Across the included evidence, several categories of empirical data are entirely absent. There are no prospective, comparator-controlled trials of AI-assisted pre-travel consultation against usual care; no datasets reporting clinically meaningful downstream outcomes such as vaccine uptake, malaria prophylaxis adherence, adverse events or travel-acquired illness; no equity-focused datasets in visiting-friends-and-relatives, migrant, older-adult, First Nations Australian or Pacific Islander travellers; no cost-effectiveness or implementation-economic data; and no published auditing data on travel-specific hallucination rates against authoritative sources such as the CDC Yellow Book [1], WHO malaria guidance [3] or ISTM advice [2]. The structure of the evidence base is therefore biased toward feasibility and design signals (Tier 1 to Tier 2) and away from outcomes and equity data, which are the categories most needed for clinical and regulatory decision-making.

***Publication bias and reporting asymmetry.*** The travel-medicine AI literature exhibits characteristic early-field publication patterns: a small number of single-site or single-tool reports with favourable framing [23,24], expert editorials advocating supervised adoption [9,10] and a design decalogue with a pre-alpha prototype [25], without offsetting null or negative implementation reports. Adjacent clinical-AI literature identifies systematic underreporting of harms, inadequate model-version documentation and heterogeneous evaluation rubrics [14,43], all of which plausibly operate in travel-medicine AI as well. Until travel-medicine AI evaluations are reported under CONSORT-AI, SPIRIT-AI and TRIPOD + AI [29,30,42,43], including registered protocols and pre-specified outcomes, publication bias should be assumed rather than excluded.

***Field maturity.*** Travel-medicine AI is at an early stage of methodological maturity. Direct evidence is dominated by a single 26-traveller implementation [24] and a single non-patient expert evaluation [23]; reporting standards specific to clinical AI [29,30,42,43] are not yet routinely applied; benchmark datasets and reference standards specific to pre-travel decision support are absent; and evaluation rubrics for hallucination, omission and outdated outbreak information in travel-medicine outputs have not been standardised. The general clinical-LLM evaluation field has only recently moved toward systematic reviews of evaluation methods [43], and travel medicine has not yet completed an equivalent stocktake.

***Regulatory and operational friction.*** No included source describes a travel-medicine AI tool that has cleared regulatory pathways as Software as a Medical Device for a travel-medicine indication under the United States FDA [33], the Australian TGA [20] or the European Union AI Act [21,22]. Operational requirements for retrieval-grounded, source-linked outputs [49], date-stamped destination data [11,12], structured intake [25], EHR integration [24,30] and ongoing hallucination auditing [12,13,14] have not been bundled into deployable products. The thinness of the evidence base therefore reflects not only research-stage immaturity but also the absence of regulated, auditable travel-medicine AI products that could generate that evidence.

### 4.3. Synthesis of Direct and Indirect Evidence

The evidence base for AI in pre-travel health consultations is asymmetric. Four direct travel-medicine AI sources [9,10,23,24,25] and one adjacent travel-related decision-support study [30] sit alongside twenty-three sources of indirect clinical AI safety, regulatory, equity and patient-engagement evidence (Tiers 3 to 5 of Table 1). This asymmetry is itself the principal finding: travel medicine currently inherits its AI evidence from adjacent specialties rather than generating it. The Singapore implementation [24] is the only Tier 1 source that involved actual travellers; the remaining direct sources are an expert evaluation of ChatGPT outputs [23], a design decalogue with a pre-alpha prototype [25] and editorial pieces framing supervised augmentation [9,10]. Inferences for clinical practice should therefore be drawn principally from Tier 1, moderated by Tier 2, and constrained—but not driven—by Tiers 3 to 5.

### 4.4. Safety Considerations

Three patterns recur across the included LLM safety literature and carry travel-medicine-specific consequences. First, hallucination and elaboration of false clinical detail are measurable and consistent across models, with planted false premises propagated in 50 to 82 percent of simulated prompts [13] and clinical guideline omission and fabrication observable in diagnostic LLM outputs [34]. Second, hallucinations are more likely than omissions to be classified as major errors in clinical documentation, particularly in plan sections [14]—a structural analogue to the recommendation-generating step of a pre-travel consultation. Third, accuracy varies widely and inconsistently across specialties, model versions and care-seeking urgency levels [35,36,43,44]. The domains where pre-travel AI is most plausibly useful—generic education, food and water safety, vaccine timing, malaria concepts [23]—are also the domains where these failure modes are least likely to cause clinically meaningful harm. The domains where AI must not act autonomously—live-vaccine eligibility, malaria prophylaxis selection, immunocompromised status clearance, drug-interaction advice, and outbreak-zone reassurance [1,3]—are precisely those where hallucination, outdated information and missing intake carry the highest harm potential. The safety risk taxonomy in Table 5 organises these failure modes against travel-medicine examples and proposed mitigations.

### 4.5. Implementation Challenges

Implementation is constrained by four practical realities. First, no commercial or open travel-medicine AI tool has regulatory approval as Software as a Medical Device for travel-medicine indications [20,21,22,33]. Second, EHR integration, retrieval-source maintenance, multilingual content review, and equity-focused assistance carry recurring cost and staffing burdens that have not been quantified in any included source [16,24,25,46]. Third, clinician adoption depends on perceived workflow fit, time savings, evidence of safety and clear allocation of liability—all of which remain unestablished for travel-medicine AI [46]. Fourth, the implementation model proposed in Table 6 and Figure 2 is a literature-informed synthesis derived from supervised-augmentation principles [9,10], design requirements [25], Singapore implementation lessons [24], FeverTravelApp adoption findings [30], retrieval-augmented generation [49] and clinician-adoption research [46]. It has not undergone external validation. Pilot deployments should be designed prospectively, with stakeholder co-design and outcomes pre-specified under CONSORT-AI or SPIRIT-AI [20,33,37], rather than retrofitted onto an unevaluated framework.

### 4.6. Ethical and Equity Issues

Ethical and equity considerations apply to every stage of the framework. WHO AI ethics guidance requires that clinical AI deployments protect autonomy, promote safety, ensure transparency and explainability, allocate responsibility and accountability, and ensure inclusiveness and equity [39]; the WHO Global Strategy on Digital Health adds equity, scalability, privacy, security and country readiness as macro-prerequisites [45]. Pre-travel populations include groups at elevated risk of both worse travel outcomes and worse access to digital tools: visiting-friends-and-relatives, migrant travellers, older adults, First Nations Australian travellers and Pacific Islander travellers face cultural, linguistic, financial and structural barriers that generic AI deployments are unlikely to mitigate and may inadvertently widen [4,5,19,32,38,48]. Direct empirical evidence on AI in these populations is absent from the included sources; this is itself a significant equity gap. Any travel-medicine AI deployment in Australia should therefore be co-designed with Aboriginal and Torres Strait Islander Community Controlled Health Services and Pacific community organisations rather than rolled out generically, and should be evaluated for cultural safety as well as accuracy [4,5,19,24,32,38,48]. Equity safeguards in Figure 2—assisted use, non-digital alternatives, multilingual and plain-language modes, cost-free access and accessibility testing—should be regarded as minimum requirements rather than optional features.

### 4.7. Future Research Priorities

Future research should prioritise five domains: (i) travel-medicine-specific hallucination audits of available chatbots benchmarked against the CDC Yellow Book 2026 [1], WHO malaria guidance [3] and ISTM advice [2], reporting accuracy, harmful omission, hallucination, citation validity and refusal behaviour [13,14,34]; (ii) prospective structured-intake trials in single travel clinics measuring consultation time, missing-data rate, clinician satisfaction and patient understanding [24,25,30]; (iii) stepped-wedge implementation trials across multiple clinics reporting vaccine uptake, malaria prophylaxis appropriateness, advice adherence and safety events under CONSORT-AI and SPIRIT-AI [20,33,37]; (iv) equity studies in older adults, low-digital-literacy travellers, visiting-friends-and-relatives travellers, First Nations Australian travellers and Pacific Islander travellers, reporting usability, completion, comprehension, preference, assisted-use need and cultural safety [4,5,19,32,38,48]; and (v) EHR-integrated retrieval-augmented systems with outbreak-feed integration, reporting recommendation concordance, auditability, privacy incidents and model drift [11,12,49]. Prediction models should follow TRIPOD + AI reporting [31], specifying discrimination, calibration, fairness and decision-curve utility. The research priority matrix in Table 9 maps these domains to immediate, near-term and longer-term horizons with suggested outcomes.

### 4.8. Practical Message for Travel Medicine Clinicians

For travel-medicine specialists, the immediate practical message is to separate low-risk information support from safety-critical decision-making. AI can explain why pre-travel consultation matters, help travellers prepare questions, and remind them how to take clinician-prescribed malaria prophylaxis [9,15,18]. AI should not independently clear a traveller for yellow fever vaccine, select malaria prophylaxis, determine live-vaccine eligibility, or reassure a high-risk traveller without clinician review [1,3,13,14,36]. Retrieval-augmented generation grounded in CDC Yellow Book, WHO malaria, and ISTM sources [1,2,3,49], combined with documented clinician oversight [9,46] and equity safeguards [32,38,48], represents the most defensible near-term architecture.

### 4.9. Supervised Implementation Model—Interpretive Synthesis

The supervised implementation model presented in Table 6 and Figure 2 (Results, Section 3.7) is an interpretive synthesis rather than an empirical finding. It integrates the supervised-augmentation principle from the Flaherty editorials [9,10], the design requirements from Baglivo et al. [25], the Singapore implementation lessons [24], the FeverTravelApp adoption findings [30], retrieval-augmented generation principles [49] and the clinician-adoption framework articulated by Scott and colleagues [46]. It is offered here as a conceptual scaffold for design, pilot and trial work, not as a validated tool, and should be tested and refined through stakeholder co-design and prospective evaluation under CONSORT-AI, SPIRIT-AI and TRIPOD + AI standards [29,30,42,43] before any clinical reliance is placed on it.

### 4.10. Regulatory Landscape—Interpretive Synthesis

The regulatory-landscape mapping in Table 7 (Results, Section 3.10) is interpretive: it translates current FDA Software as a Medical Device guidance [33], TGA medical-device software guidance [20], EU AI Act Article 6 and Annex III [21,22], WHO AI ethics guidance [39] and the WHO Global Strategy on Digital Health [45] into expected obligations for travel-medicine AI deployment. Regulatory texts evolve, and a travel-clinic deployment should obtain jurisdiction-specific advice rather than rely on the mapping presented here.

### 4.11. When Not to Use AI as the Primary Interaction—Interpretive Synthesis

Table 8 (Results, Section 3.8) lists traveller characteristics and scenarios in which AI should not be the primary interaction. This list is an interpretive synthesis of the included safety evidence [11,12,13,14,20,31,32] and authoritative travel-medicine guidance [7,8,9,19], rather than a validated triage instrument. It should be regarded as a working policy proposal to be refined through clinician co-design and prospective evaluation.

### 4.12. Research Agenda—Interpretive Synthesis

Table 9 (Results, Section 3.12) sets out a six-row research priority matrix mapping immediate, near-term, and longer-term studies to outcomes and rationale. This matrix is interpretive and reflects the gaps identified across the included evidence, particularly the absence of prospective trials [1,2,3,4,5], the absence of equity-focused studies in visiting-friends-and-relatives, migrant, First Nations Australian and Pacific Islander travellers [39,40,41,44,45,46], the absence of EHR-integrated retrieval-augmented systems for travel medicine [11,12,49] and the absence of validated travel-medicine AI prediction models [31]. Funders, journal editors and regulators have a role in shaping the field by requiring CONSORT-AI, SPIRIT-AI and TRIPOD + AI reporting [29,30,42,43] for travel-medicine AI submissions.

## 5. Limitations

This review has several important limitations.

First, the search was a transparent, targeted scoping retrieval rather than a fully reproducible multi-database systematic search. PubMed/MEDLINE, academic and web-indexed search tools, citation chasing, and authoritative guideline and regulator websites were used; searches of Embase, CINAHL, Cochrane CENTRAL, IEEE Xplore, ACM Digital Library, ClinicalTrials.gov, and WHO ICTRP and ISTM conference abstracts were planned but not run. This is a substantive limitation rather than a minor methodological caveat: omission of these databases plausibly excluded computer-science and engineering venues where travel-relevant AI prototypes and evaluations are first published (IEEE Xplore, ACM), nursing and allied-health literature documenting travel-medicine workflow innovation (CINAHL), and ongoing or unpublished trials of clinical AI in travel-relevant populations (ClinicalTrials.gov, WHO ICTRP). The included evidence base should therefore be treated as a lower bound on the field, and the central conclusions should be reassessed once a full multi-database search is conducted. Search strategies for these databases are documented in Supplement S2 as recommended reproducibility strategies but should not be presented as completed work [1,26,27,28].

Second, screening and data charting were conducted by a single reviewer, so Cohen’s kappa for inter-rater agreement is not calculable; mitigation included a structured eligibility checklist and a 24 h second-pass review (Supplement S2) [26].

Third, the PRISMA-ScR flow reflects the targeted retrieval process for this review; a future formal database run could increase the identified pool and would require updating the flow diagram (Supplement S1) [26].

Fourth, the quality appraisal is indicative rather than duplicate-assessed [40,41].

Fifth, several included sources are expert opinion, guidance, or indirect clinical AI safety evidence rather than primary pre-travel AI trials [3,4,5,6,11,12,13,14,20,31,32,33].

Sixth, formal stakeholder consultation with travel-medicine clinicians, primary care providers, regulators, traveller representatives (including visiting-friends-and-relatives travellers, older adults, First Nations Australian and Pacific Islander travellers) and digital health developers was not conducted. This is a substantive limitation that bears directly on the proposed implementation model in Table 6 and Figure 2, and it should be weighed accordingly when interpreting that model. Specifically, (i) the workflow stages (booking, waiting room, consultation, after-visit, during travel) reflect a literature-derived idealisation that may not map to real clinic capacity, staffing, scheduling or referral pathways, and may underestimate the time and governance burden of clinician sign-off at each stage; (ii) the safety safeguards and hard-stop escalation triggers were not validated against clinician workflow tolerance, alert-fatigue thresholds or local medico-legal expectations, and may be either too permissive in low-risk cases or operationally unworkable in high-volume clinics; (iii) the equity safeguards (assisted use, multilingual and plain-language modes, non-digital alternatives, cost-free access) reflect the published equity literature [39,40,41,44,45,46] rather than lived-experience input from visiting-friends-and-relatives, migrant, older-adult, First Nations Australian or Pacific Islander travellers themselves, and may therefore miss culturally specific design requirements identifiable only through co-design; and (iv) the governance and quality-assurance layer (audit, incident reporting, model-version logging, safety review board) reflects current published regulatory texts [20,21,22,23,24,25] rather than how regulators, clinic administrators and developers would actually allocate responsibility for AI failures in practice. The implementation model in Table 6 and Figure 2 is therefore a literature-based synthesis, not a co-designed framework, and should be regarded as a hypothesis to be tested and refined through formal stakeholder co-design—particularly with Aboriginal and Torres Strait Islander Community Controlled Health Services, Pacific community organisations and travel-medicine clinical leadership—before any pilot deployment. Patient and public involvement was likewise not undertaken in this review (see Patient and Public Involvement statement) and is recommended as a minimum requirement for any subsequent co-designed deployment.

Seventh, no primary cost-effectiveness or implementation-economic evidence was identified.

These limitations do not invalidate the central conclusion, but they limit certainty [42]. The review is positioned as a scoping review that maps an emerging field and proposes a safety-oriented research agenda, not as a definitive effectiveness review.

## 6. Conclusions

AI tools are likely to become part of pre-travel health workflows, but current evidence supports supervised augmentation rather than autonomous clinical decision-making [1,2,3,4,5,12,13,31,32,34]. The strongest near-term use cases are structured intake, patient education, clinician-reviewed guideline retrieval, multilingual explanation, and after-visit reinforcement [9,15,17,18,25,49]. The highest-risk use cases are vaccine contraindication screening, malaria prophylaxis selection, live-vaccine clearance, complex medication interaction advice, and reassurance of high-risk travellers [1,3,13,14,36].

Advancement of this field requires formal database searches, duplicate screening, standardised AI safety benchmarks, domain-specific hallucination audits, equity testing across VFR, migrant, older-adult, First Nations Australian, and Pacific Islander travellers, and prospective clinic trials measuring clinically meaningful outcomes, reported in line with CONSORT-AI, SPIRIT-AI, and TRIPOD + AI [29,30,42,43].

## Figures and Tables

**Table 1:** Population, Concept, and Context (PCC) framework for the scoping review.

PCC Element	Operational Definition	Examples
Population	International travellers receiving or seeking pre-travel health advice; clinicians providing pre-travel care; and study cohorts within general clinical AI safety research where the findings are mapped to pre-travel decision support	Adult and paediatric international travellers, VFR travellers, migrant travellers, immunocompromised travellers, primary care physicians, travel medicine specialists, simulated patient cohorts in clinical LLM studies
Concept	AI tools, large language models, chatbots, retrieval-augmented generation, and clinical decision-support systems applied to pre-travel risk assessment, education, intake, recommendation, escalation, or after-visit reinforcement; clinical AI safety, hallucination, and reporting standards	ChatGPT and GPT-4-based pre-travel assistants, custom GPT prototypes, tablet-based travel CDSS, generative AI educational outputs, RAG architectures, hallucination and accuracy audits
Context	International, multilingual, ambulatory pre-travel and travel-related clinical settings; primary care, specialist travel clinics, university-affiliated travel medicine services; relevant guideline, regulatory, and equity contexts	High-, middle-, and low-income settings; United States, Australia, Singapore, Switzerland, Italy, EU; CDC, WHO, ISTM guidance; FDA, TGA, EU AI Act regulatory frameworks

**Table 2:** Evidence hierarchy for the scoping review of AI tools in pre-travel health consultations, distinguishing direct travel-medicine AI evidence from indirect supporting evidence and showing the strength of inference each tier permits.

Evidence Tier	Description	Sources (n)	Examples (Reference Numbers)	Strength of Inference for Pre-Travel AI
Tier 1—Direct travel-medicine AI evidence	Empirical or design studies of AI tools applied to pre-travel health consultations or travel-medicine workflows in which the AI tool is evaluated, prototyped, or implemented.	4	Ngiam et al. ChatGPT pre-travel advice evaluation [23]; Koh et al. Singapore Travel Clinic Assistant implementation [24]; Baglivo et al. decalogue and prototype [25]; Flaherty editorials [9,10].	Supports cautious conclusions about feasibility, acceptability and design principles. Does not support efficacy or safety claims.
Tier 2—Adjacent travel-related decision-support evidence	Studies of clinical decision support applied to travel-related presentations but not to the pre-travel consultation itself.	1	Vibert et al. FeverTravelApp [30]; with Heidema et al. GeoSentinel-AI outbreak surveillance [12] treated as adjacent rather than direct.	Supports workflow and adoption lessons; does not transfer directly to pre-travel risk advice.
Tier 3—General clinical AI safety and accuracy evidence	Empirical studies of LLM accuracy, hallucination, evaluation methods and clinical guideline omission, conducted outside travel medicine.	7	Shool et al. systematic review [43]; Collins multi-model assurance [13]; Asgari et al. CREOLA framework [14]; van Kessel et al. guideline omission/hallucination [34]; Bagde et al. meta-analysis [35]; Duong et al. care-seeking accuracy [36]; Geracitano et al. ChatGPT FAQs [44].	Identifies failure modes and accuracy ranges that plausibly apply to pre-travel AI but require travel-medicine-specific replication.
Tier 4—Authoritative guidelines and regulatory texts	Clinical guidance defining the standard pre-travel consultation, plus AI and medical-device regulatory frameworks.	11	CDC Yellow Book pre-travel guidance and VFR chapter [1,19]; ISTM fact sheet [2]; WHO malaria guidance [3]; FDA SaMD, TGA, EU AI Act Articles 6 and Annex III, WHO AI ethics and Global Strategy on Digital Health [20,21,22,33,39,45]; CONSORT-AI, SPIRIT-AI, TRIPOD + AI reporting standards [20,31,33,37].	Reference standards for what AI must support and how it must be evaluated; not evidence of AI performance.
Tier 5—Adjacent implementation, equity and patient-engagement evidence	Reviews and analyses of clinical AI adoption, patient education, chatbot effectiveness, pre-travel uptake and digital equity.	12	Scott et al. clinician adoption [46]; Paranjape et al. AI in medical education [47]; Bajwa et al. healthcare AI overview [8]; Maguire et al. and Jentes et al. VFR studies [4,5]; Alotaibi et al. and Mboowa et al. primary care travel medicine [6,7]; Aydin et al. patient education LLMs [15]; Kurniawan et al. chronic-illness chatbots [16]; AHRQ healthcare chatbot review [17]; Iyer et al. preventive-care chatbot outreach [18]; digital divide and equity [32,38,48]; Peng et al. retrieval-augmented generation [49].	Supports plausibility, equity caution and design principles; does not constitute direct travel-medicine AI evidence.

Only four sources (Tier 1) directly address AI in pre-travel consultations, and only one of these—the 26-traveller Singapore implementation [24]—involved actual travellers. All other tiers inform the synthesis indirectly: they constrain what current evidence permits clinicians to conclude, but they do not substitute for prospective travel-medicine AI trials. Readers should weigh inferences according to tier and should treat Tier 1 evidence as a feasibility signal only.

**Table 3:** Evidence synthesis table for included sources with explicit GRADE-informed certainty reasoning.

Source	Country/Setting	Source Type and Sample	AI/Tool Type	Consultation Task Mapped to CDC Yellow Book Domains [1]	Main Finding	Key Safety Concern	GRADE-Informed Certainty (Reasoning) [42]
Ngiam et al. ChatGPT pre-travel advice evaluation [23]	Not patient-setting specific	Scenario-based expert evaluation; no patient sample	General-purpose ChatGPT	General advice, vaccination, malaria prophylaxis, traveller’s diarrhoea, vector avoidance	Readable and often accurate answers to common questions	Generic advice; insufficient itinerary and comorbidity personalisation	Very low (single non-patient study; high indirectness; no comparator)
Koh et al. Travel Clinic Assistant [24]	Singapore tertiary pre-travel clinic	Implementation research letter; 26 travellers	Custom GPT-4 assistant	Pre-consultation education, query elicitation, complex traveller education	Acceptable to travellers and physicians; perceived consultation focus and knowledge benefit	Small sample, self-report outcomes, digital literacy barriers, no EHR integration, hallucination risk	Very low (small single-site implementation; subjective outcomes; serious imprecision)
Baglivo et al. travel-health chatbot decalogue [25]	Italy/prototype context	Expert framework and pre-alpha custom GPT example	Custom GPT prototype	Personalisation, geolocation, multilingual support, clinic referral, EHR aspiration	Proposed ten design requirements for safe travel-health chatbots	Prototype lacks full privacy, scope control, and EHR safeguards	Very low (framework paper; no empirical outcomes)
Flaherty editorial—supervised GenAI integration [9]	International travel medicine	Expert opinion/editorial	Generative AI broadly	Pre-clinic preparation, translation, literacy tailoring, reminders	AI may support preparation and reinforce consultation learning	Must not replace individualised clinician judgement	Very low (expert opinion; non-empirical)
Flaherty and Piyaphanee natural-history editorial [10]	International travel medicine	Expert opinion/editorial	AI broadly	Risk personalisation, behaviour prediction, surveillance	Frames AI’s potential trajectory in travel medicine	Non-empirical; aspirational	Very low (expert opinion; non-empirical)
Heidema et al. GeoSentinel-AI surveillance [12]	International	Multidisciplinary editorial/perspective	Machine-learning approaches	Outbreak detection adjacent to pre-travel risk	Demonstrates concrete adjacent AI implementation pathway	Non-empirical for pre-travel decisions	Very low (perspective paper; indirect outcomes)
Vibert et al. FeverTravelApp [30]	Switzerland; returned traveller fever workflow	Case–control simulated consultations; seven physicians, three simulated patients	Tablet clinical decision-support algorithm	Travel-related risk intake, exposure history, dynamic clinical reasoning	Demonstrates feasibility issues for travel-related CDSS in consultations	Indirect to pre-travel prevention; clinician interaction and adoption matter	Low (small simulation study; indirect to pre-travel)
CDC Yellow Book pre-travel consultation guidance [1]	United States guidance	Clinical guidance	Not AI	Gold-standard task taxonomy for pre-travel risk assessment	Defines domains AI must support and not oversimplify	Authoritative reference standard against which AI outputs should be checked; risk that AI outputs may diverge from current guidance	Not applicable (guideline; serves as reference standard)
WHO malaria travel guidance [3]	Global guidance	Clinical guidance	Not AI	Malaria geography, chemoprophylaxis, mosquito protection	Defines high-risk domain requiring up-to-date recommendations	Updates frequently; AI tools relying on training-data snapshots may be outdated	Not applicable (guideline; serves as reference standard)
ISTM pre-travel health advice [2]	International travel medicine	Professional fact sheet	Not AI	Risk assessment, timing, vaccines, medicines, chronic illness	Reinforces that travel advice extends beyond vaccines	Useful patient-facing standard; AI must not understate timing-of-consultation criticality	Not applicable (guideline; serves as reference standard)
Shool et al. LLM evaluation systematic review [43]	General clinical medicine	Systematic review; 761 studies	LLMs	Evaluation standards for clinical AI	Evaluation methods remain heterogeneous	Indirect to travel medicine	Low (systematic review; indirect outcomes)
Collins multi-model hallucination assurance [13]	General clinical decision support	Simulation study; multiple models	Multiple LLMs	Safety testing for clinical decision support	LLMs repeated or elaborated false clinical details in 50 to 82 percent of outputs	Directly relevant to hallucination risk	Moderate for general LLM risk; indirect for travel (consistent finding across multiple models)
Asgari et al. CREOLA hallucination framework [14]	Clinical documentation	Framework and evaluation study	LLMs	Clinical documentation accuracy	Hallucinations more often “major” than omissions, especially in plan sections	Directly relevant to AI-generated travel advice	Low to moderate (single framework study; high indirectness to travel)
van Kessel et al. clinical guideline hallucination analysis [34]	Clinical decision support	Diagnostic LLM systematic analysis	LLMs	Hallucination of authoritative guidelines	Identifies measurable prevalence of fabricated and omitted clinical guideline content	Highly relevant to fabricated travel-vaccine or malaria guidance	Low (single multi-model analysis; indirect to travel)
Bagde et al. ChatGPT meta-analysis [35]	Medical and dental research	Systematic review and meta-analysis	ChatGPT	Domain-specific accuracy	Accuracy 18 to 100 percent across specialties; high variability	Indirect; supports specialty-specific benchmarks	Low (high inconsistency; indirectness)
Duong et al. care-seeking accuracy [36]	General	Multi-version evaluation; 22 model versions	ChatGPT	Care-seeking advice across urgency levels	Average accuracy ~70 percent; overtriage; increasing variability with newer models	Directly supports conservative governance	Low to moderate (multi-version simulation; indirect to travel)
Geracitano et al. ChatGPT FAQ literature review [44]	General	Literature review; nine studies	ChatGPT	FAQ, recommendation, symptom categorisation	Accuracy 20 to 95 percent; not standalone point-of-care	Supports human oversight requirement	Low (small literature review; indirect to travel)
Aydin et al. patient-education LLM scoping review [15]	General medicine	Scoping review	LLMs	Patient education and engagement	LLMs may generate education material but face accuracy, readability, and bias challenges	Indirect relevance to pre-travel education	Low (scoping review; indirect outcomes)
Kurniawan et al. chronic-illness chatbot review [16]	Chronic disease management	Systematic review	Chatbots	Acceptability and effectiveness	Acceptability promising; efficacy evidence limited; insufficient technical documentation	Mirrors travel-medicine implementation gap	Low (systematic review; indirect to travel)
Iyer et al. preventive-care chatbot outreach [18]	US value-based care	Retrospective analysis	Chatbot outreach	Preventive care compliance	Chatbots underperformed phone calls overall but outperformed for diabetes care in 2023	Selective and context-dependent efficacy	Low (single retrospective analysis; indirect to travel)
Peng et al. retrieval-augmented generation [49]	Multilingual medical	Comparative evaluation; 10 LLMs	RAG architectures	Source-grounded medical answer generation	RAG improves accuracy and generalises to unseen medical languages	Supports RAG as design principle	Low to moderate (comparative empirical study; indirect to travel)

**Table 4:** Quality and applicability appraisal of included sources, with rationale and domain.

Source Category	Appraisal Approach	Domain Assessed	Rationale and Main Appraisal Judgement	Implication for Synthesis
Singapore Travel Clinic Assistant [24]	MMAT-informed implementation appraisal [40]	Sampling, outcome ascertainment, conflict-of-interest control	Single centre, 26 travellers, qualitative feedback, no comparator; no objective effectiveness outcome; selection bias possible	Useful feasibility signal, not effectiveness evidence
ChatGPT pre-travel advice evaluation [23]	Custom accuracy-study appraisal	Scenario coverage, reproducibility, expert benchmarking	Clinically relevant questions and expert comparison; no patient outcomes; no model-version reproducibility; limited scenario diversity	Supports educational potential only
Decalogue and editorials [3,4,5,6]	JBI text/opinion-informed appraisal [29]	Domain expertise, logical consistency, relevance	Strong domain expertise and clinical logic but non-empirical and prescriptive rather than evaluative	Useful for implementation principles, not outcome claims
FeverTravelApp [30]	MMAT-informed appraisal [40]	Study design, sample size, blinding, comparator	Empirical travel-related CDSS evidence but post-travel and simulated; small physician sample; indirect to pre-travel	Useful for workflow and adoption lessons
General clinical LLM systematic review [43]	AMSTAR 2-informed appraisal [41]	PRISMA reporting, search comprehensiveness, risk of bias assessment	Large and relevant review, transparent methods, but indirect to travel medicine; heterogeneity not pooled	Supports need for standardised evaluation
Multi-model hallucination, accuracy, and guideline-hallucination studies [12,13,14,20,31,32]	Simulation- and review-study appraisal	Reproducibility, prompt control, clinical reference standard	Strong safety signal for LLM vulnerability and accuracy variability; generally not travel-specific; consistent across models in [13]	Supports conservative governance and human review
Patient-education and chatbot reviews [15,16,17,18]	AMSTAR 2-informed appraisal [41]	Search, selection, synthesis transparency	Relevant adjacent evidence; heterogeneous designs and outcomes; limited primary trial data	Supports plausibility of supervised AI use and equity caution
Guidelines and authoritative texts [7,8,9,19,39]	JBI text-and-opinion-informed appraisal [29]	Authority, currency, scope	Authoritative guidance from CDC, WHO, ISTM; not designed as evidence appraisal targets but as reference standards	Used as the gold-standard task taxonomy and reference standard, not as outcome evidence

**Table 5:** Clinical safety risk taxonomy for AI in pre-travel consultations.

AI Failure Mode	Travel Medicine Example	Potential Patient Safety Consequence	Mitigation
False destination risk claim [3,13,34]	Incorrectly states that a specific region has no malaria risk	Omitted chemoprophylaxis or inadequate mosquito precautions	Retrieval-grounded malaria source [3,49], date-stamped destination data, clinician review
Outdated outbreak information [11,12,34]	Misses active yellow fever, polio, measles, dengue, or mpox advisory	Unvaccinated or underprepared traveller enters risk zone	Real-time public health feed, source timestamp, no model-memory-only outbreak advice
Contraindication miss [1,14]	Recommends live vaccine to immunocompromised traveller	Vaccine-derived illness or serious adverse event	Mandatory immune-status questions and hard-stop clinician review
Drug interaction error [1,14]	Ignores psychiatric history or interacting medication when discussing mefloquine	Neuropsychiatric adverse event or poor adherence	Medication reconciliation, contraindication checklist, pharmacist or clinician sign-off
False reassurance [13,36]	Tells a splenectomy patient that malaria risk is routine or low	Life-threatening malaria risk underestimated	High-risk condition trigger and escalation to specialist review
Incomplete history intake [1,25]	Does not ask about pregnancy, transplant, HIV, anticoagulation, allergy, prior vaccines, or itinerary details	Inappropriate vaccine, medication, or risk counselling	Structured intake before any recommendation
Hallucinated authority [13,14,34,44]	Fabricates a guideline, dose, requirement, or clinic policy	Clinician or patient follows non-existent recommendation	Source-linked output only [49]; block unsupported claims
Equity failure [4,5,24,32,38,48]	Older-adult or low-literacy traveller cannot use tool	Exclusion of high-risk groups from pre-consultation support	Assisted use, multilingual and plain-language modes, non-digital alternative
Overtriage or undertriage [36]	Routes low-acuity question to emergency or vice versa	Resource misuse or delayed care	Calibrated triage thresholds, clinician review of escalations

***Caveat.*** The safety risk taxonomy in Table 5 is a literature-informed conceptual framework synthesised from the included LLM safety, hallucination and accuracy literature [13,14,34,35,36,43,44]; travel-medicine guidance [1,2,3,11]; and the empirical travel-medicine implementations [23,24,30]. It has not undergone external validation in prospective travel-medicine deployments. Each failure mode and mitigation should be regarded as a hypothesis to be tested under CONSORT-AI, SPIRIT-AI and TRIPOD + AI standards before any clinical reliance is placed on it [20,31,33,37].

**Table 6:** Supervised AI implementation model for pre-travel consultation workflows.

Workflow Stage	What AI Does at This Stage	Triggers That Require Clinician Handover	Required Safeguards and Clinic Policy	Who is Responsible
Booking (before the visit)	Captures the traveller’s itinerary, departure date, destinations, planned activities, and baseline medical and medication history [1,25].	Send to clinician now if the traveller is pregnant, immunosuppressed, a transplant or HIV patient, has had a splenectomy, has a complex multi-country itinerary, or is departing within 14 days.	Display a privacy notice; collect only the minimum data needed; audit weekly that intake forms are complete [39,45].	Clinic lead
Waiting room (just before the consultation)	Delivers general travel-health education and elicits questions the traveller wants to raise with the clinician [9,15,17].	Stop the AI conversation and route to a clinician if the traveller asks for a vaccine clearance, a medication prescription, a diagnosis, or any high-risk advice.	AI runs in source-grounded education mode only; no prescribing, dispensing, or definitive clinical advice is permitted [49].	Travel clinician
Consultation (with the clinician)	Generates a structured summary of risks, missing data, and relevant guideline prompts to support the clinician’s decision [25,30,46].	Flag for clinician review if vaccine history is incomplete, immune status is unclear, a drug interaction is possible, or the traveller raises a live-vaccine question.	The clinician must verify every AI recommendation before acting on it; the final plan is the clinician’s, not the model’s [9,14].	Travel clinician
After-visit (follow-up messaging)	Reinforces the clinician-approved plan, vaccine schedule, malaria chemoprophylaxis instructions, and behavioural advice [9,18].	Reconnect the traveller with the clinic when they report an adverse reaction, fever, new pregnancy, itinerary change, or medication intolerance.	All follow-up content has been pre-approved by a clinician; a documented escalation pathway connects the traveller back to the clinic.	Clinic protocol owner
During travel (in-country support)	Provides emergency contact numbers, scheduled reminders, and red-flag warnings while the traveller is abroad [3,9].	Direct the traveller to seek in-person care for fever after malaria exposure, animal bite, severe diarrhoea, respiratory distress, sexual exposure, or significant injury.	AI must give immediate seek-care advice for any red flag and must never attempt autonomous diagnosis.	Traveller support protocol
Quality assurance (ongoing oversight)	Continuously audits AI outputs and user feedback to detect failures over time [13,14,34,43,46].	Investigate immediately if an audit detects a hallucinated recommendation, an outdated source, an unsafe omission, or an inequitable use pattern.	Run a monthly random-sample audit; maintain an incident register and a model-version log; convene a safety review board [20,33,39].	Clinical governance committee

**Table 7:** Regulatory landscape for AI in pre-travel health consultations.

Jurisdiction	Regulatory Signal	Relevance to Travel-Medicine AI
United States FDA [33]	FDA describes AI/ML in Software as a Medical Device as requiring lifecycle management and appropriate premarket pathways such as 510(k), De Novo, or premarket approval, depending on intended use	A tool that merely educates may be lower risk, while a tool that drives vaccine or medication recommendations may approach regulated clinical decision support
Australia TGA [20]	TGA regulates software when it meets the medical-device definition under section 41BD of the Therapeutic Goods Act 1989, and developers of AI-enabled medical device software may be manufacturers or sponsors	Australian travel clinics should assess intended use, claims, risk class, sponsor obligations, and post-market monitoring before deploying AI decision tools
European Union AI Act Article 6 [21]	Article 6 classifies AI as high-risk when it meets product safety conditions or falls within Annex III categories, with exemptions only where it does not pose significant risk to health, safety, or fundamental rights	Travel AI affecting health decisions may require high-risk analysis, especially if it materially influences clinical recommendations
European Union AI Act Annex III [22]	Annex III includes systems related to healthcare service eligibility, health insurance risk assessment, emergency healthcare triage, and health-risk assessment in migration/border contexts	A travel-health AI tool handling triage or health-risk classification should be assessed for high-risk obligations and documentation requirements
WHO AI ethics guidance [39]	Six consensus principles: Protect autonomy, promote human well-being and safety, ensure transparency and explainability, foster responsibility and accountability, ensure inclusiveness and equity, and promote responsive and sustainable AI	Travel-medicine AI deployment should map governance to these principles
WHO Global Strategy on Digital Health [45]	Emphasises equity, scalability, privacy, security, and country readiness as prerequisites for digital health deployment	Travel-medicine AI should be evaluated against these macro-level prerequisites

**Table 8:** When not to use AI as the primary interaction.

Traveller Characteristic	Risk Category	Required Action
Pregnancy or planning pregnancy [1,3]	Live-vaccine and antimalarial contraindication risk	Mandatory clinician review; AI restricted to information-gathering
Immunocompromised (transplant, advanced HIV, immunosuppressive therapy, asplenia) [1]	Vaccine-derived illness and severe travel infection risk	Mandatory specialist review; AI must not advise on vaccine eligibility
Anticoagulation or unstable cardiovascular disease [1]	Drug-interaction and travel-stress risk	Clinician review of medication and itinerary
Severe allergy or anaphylaxis history [1]	Vaccine reaction risk	Clinician-led vaccine selection and observation planning
Complex psychiatric history [1]	Mefloquine and other neuropsychiatric medication risks	Clinician-led prophylaxis selection
Travel within two weeks [2]	Inadequate time for vaccine schedules	Triaged clinician review and accelerated schedule
Outbreak-zone travel [3,11,12]	Time-sensitive epidemiology beyond model knowledge	Real-time public health source and clinician review
Live vaccine clearance request	Direct contraindication assessment	Clinician-only decision
Malaria prophylaxis selection request [3]	Resistance, drug-interaction, comorbidity-specific decision	Clinician-only prescribing
Post-exposure care after animal bite, sexual exposure, or needlestick	Time-critical post-exposure prophylaxis	Direct clinician contact, emergency services if needed
Severe digital literacy or language barriers [4,5,24,32,38,48]	Equity and comprehension risk	Assisted use and non-digital alternative
First Nations Australian or Pacific Islander traveller in absence of culturally adapted content [4,5,19,32,38,48]	Cultural safety and trust	Co-designed clinician pathway; not generic AI as primary interaction

**Table 9:** Research priority matrix for AI in pre-travel health consultations.

Priority	Study or Activity	Rationale	Suggested Outcomes
Immediate	Hallucination audit of travel-medicine chatbots against CDC Yellow Book 2026 [1], WHO malaria guidance [3], and ISTM advice [2]	High urgency and feasible with simulated cases [13,14,34]	Accuracy, harmful omission, hallucination, citation validity, refusal behaviour
Immediate	Prospective structured intake trial in one travel clinic [24,25,30]	High feasibility and direct workflow relevance	Consultation time, missing-data rate, clinician satisfaction, patient understanding
Near-term	Stepped-wedge trial across multiple travel clinics following CONSORT-AI/SPIRIT-AI [20,33,37]	Tests implementation under real-world variation	Vaccine uptake, malaria prophylaxis appropriateness, advice adherence, safety events
Near-term	Equity study in older adults, low-digital-literacy travellers, VFR travellers, First Nations Australians, and Pacific Islander travellers [4,5,19,32,38,48]	Addresses likely access asymmetry	Usability, completion, comprehension, preference, assisted-use need, cultural safety
Longer-term	EHR-integrated retrieval-augmented generation system with outbreak-feed integration [11,12,49]	Highest potential but greater regulatory and privacy burden [20,21,22,33]	Recommendation concordance, auditability, privacy incidents, model drift
Longer-term	Multilingual validation across common traveller origin languages [49]	Needed for global travel medicine	Translation fidelity, cultural appropriateness, safety equivalence
Longer-term	Travel-medicine AI prediction models for risk stratification, reported per TRIPOD + AI [31]	Enables individualised pre-travel risk advice	Discrimination, calibration, fairness, decision-curve utility

## Data Availability

Search files, extracted source summaries, the PRISMA-ScR figure [26], the completed PRISMA-ScR checklist (Supplement S1), the search strategy and eligibility checklist (Supplement S2), the excluded and retrieval log and characteristics-of-included-sources tables (Supplements S3 and S4), and the minimum reporting standards checklist and tool inventory (Supplement S5) accompany this manuscript. A formal future submission should include database-specific search histories and an excluded-studies table extended through additional databases.

## Supplementary Materials

The following supporting information can be downloaded at: https://www.mdpi.com/article/10.3390/tropicalmed11070186/s1, Supplement S1—Completed PRISMA-ScR checklist; Supplement S2—Search strategy and eligibility checklist; Supplement S3—Excluded records and retrieval log; Supplement S4—Characteristics of included sources; Supplement S5—Minimum reporting standards checklist and tool inventory.
