# Supplementary material for: Artificial Intelligence Tools in Pre-Travel Health Consultations: A Scoping Review of Clinical Evidence, Implementation Gaps, and Emerging Opportunities
Source: Trop Med Infect Dis. 2026 Jul 6;11(7):186. doi: 10.3390/tropicalmed11070186 (PMC13431339; doi:10.3390/tropicalmed11070186)
Supplement: Supplementary file 1 [file tropicalmed-11-00186-s001.zip › Supplement_S1_PRISMA-ScR_Checklist.pdf]

Supplementary Material — Supplement S1

Completed PRISMA-ScR Checklist

Artificial Intelligence Tools in Pre-Travel Health Consultations: A Scoping Review of Clinical Evidence, Implementation Gaps, and Emerging Opportunities

Haider Saddam Qasim (corresponding author) and Maree Donna Simpson · Tropical Medicine and Infectious Disease, MDPI · 2026

This supplement provides the completed PRISMA-ScR (Preferred Reporting Items for Systematic reviews and Meta-Analyses extension for Scoping Reviews) 22-item checklist, cross-referenced to the corresponding sections of the manuscript. The checklist follows Tricco et al. (2018) as recommended by the Joanna Briggs Institute scoping review guidance.

Section	Item	PRISMA-ScR Item Name	Checklist Item Description	Reported in Manuscript
TITLE	1	Title	Identify the report as a scoping review.	Title page (identifies the work as “A Scoping Review …”).
ABSTRACT	2	Structured summary	Provide a structured summary that includes (as applicable) background, objectives, eligibility criteria, sources of evidence, charting methods, results, and conclusions.	Abstract.
INTRODUCTION	3	Rationale	Describe the rationale for the review in the context of what is already known. Explain why the review questions/objectives lend themselves to a scoping review approach.	Section 1 — Introduction.
INTRODUCTION	4	Objectives	Provide an explicit statement of the questions and objectives being addressed with reference to their key elements (e.g., population or participants, concepts, and context) or other relevant key elements used to conceptualize the review questions and/or objectives.	Section 1 — Introduction (aim statement) and Section 2.2 — PCC framework.
METHODS	5	Protocol and registration	Indicate whether a review protocol exists; state if and where it can be accessed (e.g., a Web address); and if available, provide registration information, including the registration number.	Section 2.1 — Review Design (states no prospective registration; no protocol published).
METHODS	6	Eligibility criteria	Specify characteristics of the sources of evidence used as eligibility criteria (e.g., years considered, language, and publication status), and provide a rationale.	Section 2.3 — Eligibility Criteria; Supplement S2.
METHODS	7	Information sources	Describe all information sources in the search (e.g., databases with dates of coverage and contact with authors to identify	Section 2.4 — Search Strategy; Supplement S2.

Section	Item	PRISMA-ScR Item Name	Checklist Item Description	Reported in Manuscript
			additional sources), as well as the date the most recent search was executed.	
METHODS	8	Search	Present the full electronic search strategy for at least one database, including any limits used, such that it could be repeated.	Section 2.4 — PubMed/MEDLINE search string reproduced in full; Supplement S2 — full strategy and planned reproducibility strings for Embase, CINAHL, Cochrane CENTRAL, IEEE Xplore, ACM Digital Library, ClinicalTrials.gov, WHO ICTRP.
METHODS	9	Selection of sources of evidence	State the process for selecting sources of evidence (i.e., screening and eligibility) included in the scoping review.	Section 2.5 — Study Selection and Data Charting; Section 2.7 — Study Selection Flow; Supplement S2 — eligibility checklist.
METHODS	10	Data charting process	Describe the methods of charting data from the included sources of evidence (e.g., calibrated forms or forms that have been tested by the team before their use, and whether data charting was done independently or in duplicate) and any processes for obtaining and confirming data from investigators.	Section 2.5 — Study Selection and Data Charting; Supplement S4 — completed charting table.
METHODS	11	Data items	List and define all variables for which data were sought and any assumptions and simplifications made.	Section 2.5 — Study Selection and Data Charting (charted fields enumerated).
METHODS	12	Critical appraisal of individual sources of evidence	If done, provide a rationale for conducting a critical appraisal of included sources of evidence; describe the methods used and how this information was used in any data synthesis (if appropriate).	Section 2.6 — Quality Appraisal and Certainty Assessment; Section 3.4 — Quality and Applicability Appraisal (Table 4); Supplement S5 — minimum reporting standards checklist.
METHODS	13	Synthesis of results	Describe the methods of handling and summarizing the data that were charted.	Section 2.5 — Data Charting; Section 3 — Results (tiered evidence synthesis; Tables 2, 3, 4).
RESULTS	14	Selection of sources of evidence	Give numbers of sources of evidence screened, assessed for eligibility, and included in the review, with reasons for exclusions at each stage, ideally using a flow diagram.	Section 2.7 — Study Selection Flow and Figure 1 (PRISMA-ScR flow diagram); Supplement S3 — excluded records log.

Section	Item	PRISMA-ScR Item Name	Checklist Item Description	Reported in Manuscript
RESULTS	15	Characteristics of sources of evidence	For each source of evidence, present characteristics for which data were charted and provide the citations.	Section 3 — Results; Tables 2 and 3; Supplement S4 — extended characteristics table.
RESULTS	16	Critical appraisal within sources of evidence	If done, present data on critical appraisal of included sources of evidence (see item 12).	Section 3.4 — Quality and Applicability Appraisal; Table 4.
RESULTS	17	Results of individual sources of evidence	For each included source of evidence, present the relevant data that were charted that relate to the review questions and objectives.	Table 3 — Evidence synthesis table with per-source findings, safety concerns, and GRADE-informed certainty.
RESULTS	18	Synthesis of results	Summarize and/or present the charting results as they relate to the review questions and objectives.	Section 3.1–3.3 — tiered synthesis narrative; Table 3.
DISCUSSION	19	Summary of evidence	Summarize the main results (including an overview of concepts, themes, and types of evidence available), link to the review questions and objectives, and consider the relevance to key groups.	Section 4 — Discussion (opening subsections; principal findings).
DISCUSSION	20	Limitations	Discuss the limitations of the scoping review process.	Section 4 — Discussion (limitations subsection); Section 2.4 — Search Strategy (databases not executed acknowledged).
DISCUSSION	21	Conclusions	Provide a general interpretation of the results with respect to the review questions and objectives, as well as potential implications and/or next steps.	Section 5 — Conclusions.
FUNDING	22	Funding	Describe sources of funding for the included sources of evidence, as well as sources of funding for the scoping review. Describe the role of the funders of the scoping review.	Funding statement (no funding received); Conflicts of Interest statement.

Reference

Tricco, A.C.; Lillie, E.; Zarin, W.; O'Brien, K.K.; Colquhoun, H.; Levac, D.; Moher, D.; Peters, M.D.J.; Horsley, T.; Weeks, L.; et al. PRISMA Extension for Scoping Reviews (PRISMA-ScR): Checklist and Explanation. Ann. Intern. Med. 2018, 169, 467–473. doi:10.7326/M18-0850.
