# Supplementary material for: Artificial Intelligence Tools in Pre-Travel Health Consultations: A Scoping Review of Clinical Evidence, Implementation Gaps, and Emerging Opportunities
Source: Trop Med Infect Dis. 2026 Jul 6;11(7):186. doi: 10.3390/tropicalmed11070186 (PMC13431339; doi:10.3390/tropicalmed11070186)
Supplement: Supplementary file 1 [file tropicalmed-11-00186-s001.zip › Supplement_S2_Search_Strategy_and_Eligibility.pdf]

Supplementary Material — Supplement S2

Search Strategy and Eligibility Checklist

Artificial Intelligence Tools in Pre-Travel Health Consultations: A Scoping Review of Clinical Evidence, Implementation Gaps, and Emerging Opportunities

Haider Saddam Qasim (corresponding author) and Maree Donna Simpson · Tropical Medicine and Infectious Disease, MDPI · 2026

This supplement documents the search strategy, database-specific search strings (executed and planned), the PCC-anchored eligibility checklist, and the decision rules used during single-reviewer screening. It corresponds to Sections 2.3–2.5 of the manuscript.

S1. Search Overview

This scoping review used a transparent, targeted retrieval rather than a fully reproducible multi-database systematic search. Retrieval was conducted in May 2026 covering January 2017 to May 2026. One direct PubMed/MEDLINE search string was executed and is reproduced verbatim below. Complementary strings for Embase, CINAHL, Cochrane CENTRAL, IEEE Xplore, ACM Digital Library, ClinicalTrials.gov, WHO ICTRP, and ISTM conference abstracts were prepared as reproducibility-ready strategies but were not executed within this scoping retrieval. This restriction is recorded transparently as a methodological limitation and is a priority for any future confirmatory review.

S2. Executed Search — PubMed/MEDLINE (May 2026)

Interface: PubMed / MEDLINE. Executed: May 2026. Date range applied: 2017/01/01 to 2026/05/31 (Publication Date filter). Language filter: English.

Search string (reproduced verbatim):

("travel medicine"[Title/Abstract] OR "pre-travel"[Title/Abstract] OR "pretravel"[Title/Abstract] OR "travel health"[Title/Abstract] OR "traveller health"[Title/Abstract]) AND ("artificial intelligence"[Title/Abstract] OR "large language model"[Title/Abstract] OR "ChatGPT"[Title/Abstract] OR chatbot*[Title/Abstract] OR "clinical decision support"[Title/Abstract] OR "machine learning"[Title/Abstract])

Records identified: 70 (PubMed/MEDLINE combined with citation chasing on the four direct travel-medicine AI sources and the FeverTravelApp study, plus targeted retrieval from CDC, WHO, ISTM, FDA, TGA, and the European Commission / EU AI Act portal). After removal of one duplicate, 69 records were screened.

S3. Planned Reproducibility Strings (Not Executed in This Retrieval)

The following database-specific translations are recommended for any subsequent confirmatory review. They are provided so that the strategy is fully reproducible by an independent reviewer.

Database / Interface	Search String (planned)
Embase (Elsevier / Emtree)	('travel medicine'/exp OR 'pre-travel':ti,ab,kw OR 'pretravel':ti,ab,kw OR 'travel health':ti,ab,kw) AND ('artificial intelligence'/exp OR 'large language model':ti,ab,kw OR 'ChatGPT':ti,ab,kw OR 'chatbot*':ti,ab,kw OR 'clinical decision support system'/exp OR 'machine learning'/exp) AND [2017-2026]/py

Database / Interface	Search String (planned)
CINAHL Complete (EBSCOhost)	((MH "Travel Medicine+") OR TI ("pre-travel" OR "pretravel" OR "travel health") OR AB ("pre-travel" OR "pretravel" OR "travel health")) AND ((MH "Artificial Intelligence+") OR TI ("large language model" OR "ChatGPT" OR chatbot* OR "clinical decision support" OR "machine learning") OR AB ("large language model" OR "ChatGPT" OR chatbot* OR "clinical decision support" OR "machine learning")) Limiters: Publication Year 2017–2026.
Cochrane CENTRAL (Wiley CDSR/CENTRAL Advanced Search)	("travel medicine" OR "pre-travel" OR pretravel OR "travel health") AND ("artificial intelligence" OR "large language model" OR ChatGPT OR chatbot* OR "clinical decision support" OR "machine learning") in Title Abstract Keyword — with Publication Year from 2017 to 2026, in Trials.
IEEE Xplore (Command Search)	(("All Metadata":"travel medicine" OR "All Metadata":"pre-travel" OR "All Metadata":pretravel OR "All Metadata":"travel health") AND ("All Metadata":"artificial intelligence" OR "All Metadata":"large language model" OR "All Metadata":ChatGPT OR "All Metadata":chatbot* OR "All Metadata":"clinical decision support" OR "All Metadata":"machine learning")) Publication Year: 2017–2026.
ACM Digital Library (Advanced Search)	[All: "travel medicine"] OR [All: "pre-travel"] OR [All: pretravel] OR [All: "travel health"] AND [All: "artificial intelligence"] OR [All: "large language model"] OR [All: ChatGPT] OR [All: chatbot*] OR [All: "clinical decision support"] OR [All: "machine learning"] Publication Date: 2017–2026.
ClinicalTrials.gov (Advanced Search)	Condition or disease: “travel” OR “pre-travel” OR “traveller health”; Other terms: “artificial intelligence” OR “large language model” OR “ChatGPT” OR “chatbot” OR “clinical decision support”. Study Start: 01/01/2017 to 31/05/2026.
WHO ICTRP	Search text: (travel medicine OR pre-travel OR travel health) AND (artificial intelligence OR large language model OR ChatGPT OR chatbot OR clinical decision support). Recruitment status: all. Date of registration: 01-01-2017 to 31-05-2026.
ISTM Conference Abstracts / Journal of Travel Medicine hand-search	Manual scan of Journal of Travel Medicine issues (2017–May 2026), CISTM/NECTM/APTHC conference abstract books for titles containing: artificial intelligence, LLM, ChatGPT, chatbot, clinical decision support, or machine learning.

S4. Grey Literature and Hand-Search Sources

- Journal of Travel Medicine (Oxford University Press) — table-of-contents scan, 2017 to May 2026.
- Travel Medicine and Infectious Disease (Elsevier) — table-of-contents scan, 2017 to May 2026.
- BMC Digital Health and Communications Medicine — AI-relevant titles within the search window.
- CDC Yellow Book 2026 (chapter-level retrieval), ISTM website, WHO International Travel and Health, WHO Digital Health Strategy 2020–2025.
- Regulator documents: FDA (Software as a Medical Device), Therapeutic Goods Administration (Software-Based Medical Devices), European Commission / EU AI Act portal.
- Citation chasing on the four direct travel-medicine AI sources and the FeverTravelApp study.

S5. Eligibility Checklist — PCC Framework

The following checklist was applied to every retrieved record at title-and-abstract screening and again at full-text assessment. A record was eligible only when it satisfied at least one Population, one Concept, and one Context row without triggering any exclusion criterion.

Domain	Include if…	Exclude if…
Population (P)	International travellers (adult and paediatric; migrant, VFR, immunocompromised); clinicians providing pre-travel care; simulated patient cohorts in clinical LLM studies where findings map to pre-travel decision support.	Studies of non-travel populations with no explicit or transferable link to pre-travel decision support.
Concept (C)	AI tools, large language models, chatbots, retrieval-augmented generation, or clinical decision-support systems applied to pre-travel risk assessment, education, intake, recommendation, escalation, or after-visit reinforcement; clinical AI safety, hallucination, and reporting standards where findings map to pre-travel care.	Non-AI interventions; purely technical AI/ML papers without clinical implementation or evaluation content; non-clinical AI (marketing chatbots, hospitality bots, education AI).
Context (Ctx)	International, multilingual, ambulatory pre-travel and travel-related clinical settings; primary care and specialist travel clinics; university-affiliated travel medicine services; relevant guideline, regulatory (FDA, TGA, EU AI Act), and equity contexts.	Non-clinical or general-purpose contexts unrelated to travel medicine.
Publication types included	Empirical studies (RCT, cohort, case–control, cross-sectional, mixed methods, qualitative); systematic and scoping reviews; framework and design papers; expert opinion and editorials where they inform implementation, safety, or equity; authoritative guidelines and regulator documents.	Preprints subsequently superseded by a published version; duplicate coverage of an already-included source; opinion pieces not addressing AI in or near a pre-travel consultation context.
Language	English full text or professional English translation available.	Sources without accessible English full text within the search window.
Time window	January 2017 to May 2026 (covering the emergence of contemporary transformer-based LLMs and the ChatGPT release).	Sources published before January 2017 unless they represent foundational travel-medicine guidance still current in the search window (e.g., CDC Yellow Book chapters).
Retrievability	Full text obtainable within the May 2026 search window through institutional subscriptions, open-access repositories, or direct request.	Full text not retrievable within the search window (recorded in retrieval log — see Supplement S3).

S6. Decision Rules for Ambiguous Cases

Because screening was conducted by a single reviewer, Cohen's kappa for inter-rater agreement was not calculable. The following explicit decision rules were pre-specified and applied during screening; ambiguous records were held for second-pass review at least 24 h after the first pass.

Rule	Application
Rule 1 — Boundary of "clinical AI safety" evidence	General-medicine LLM safety, hallucination, or reporting-standards evidence is eligible only if the finding is directly transferable to pre-travel decision support (e.g., fabrication of authoritative guidelines, hallucinated dose or contraindication content). Otherwise excluded under category (iv).
Rule 2 — Boundary of "adjacent chatbot" evidence	Chronic-illness or preventive-care chatbot evidence is eligible when it illuminates acceptability, effectiveness, or equity mechanisms that also apply to travel-medicine chatbots. Excluded when confined to disease-specific outcomes with no transferable implementation lesson.
Rule 3 — Editorial / expert opinion	Editorials and opinion pieces are eligible only when they explicitly address AI in, or immediately adjacent to, a pre-travel consultation context (per JBI text-and-opinion guidance). Otherwise excluded under category (vi).
Rule 4 — Duplicate coverage	Where a preprint and a peer-reviewed version report the same dataset, only the peer-reviewed published version is included. The superseded preprint is recorded as excluded under category (v).
Rule 5 — Guideline currency	Where a guideline exists in multiple editions, the most recent edition current in the search window is included; superseded editions are noted but not double-counted.
Rule 6 — Ambiguous cases	Records that could not be unambiguously classified were held for second-pass review by the same reviewer at least 24 h after the first pass, using the written eligibility checklist. Final decisions were recorded with the rule number that resolved the ambiguity.

S7. Screening Workflow

- Step 1 — De-duplication of the 70 identified records (one duplicate removed; 69 remaining).
- Step 2 — Title-and-abstract screening against the PCC eligibility checklist by a single reviewer.
- Step 3 — Post hoc classification of the 57 excluded records into six mutually exclusive exclusion categories (see Supplement S3).
- Step 4 — Full-text retrieval sought for 12 reports; one report could not be retrieved within the search window (see Supplement S3).
- Step 5 — Full-text assessment of 11 reports against the eligibility checklist; all 11 met inclusion.
- Step 6 — Second-pass review of borderline records by the same reviewer at least 24 h after the first pass, with any changed decisions logged.
