# Supplementary material for: Artificial Intelligence Tools in Pre-Travel Health Consultations: A Scoping Review of Clinical Evidence, Implementation Gaps, and Emerging Opportunities
Source: Trop Med Infect Dis. 2026 Jul 6;11(7):186. doi: 10.3390/tropicalmed11070186 (PMC13431339; doi:10.3390/tropicalmed11070186)
Supplement: Supplementary file 1 [file tropicalmed-11-00186-s001.zip › Supplement_S3_Excluded_Records_and_Retrieval_Log.pdf]

Supplementary Material — Supplement S3

Excluded Records and Retrieval Log

Artificial Intelligence Tools in Pre-Travel Health Consultations: A Scoping Review of Clinical Evidence, Implementation Gaps, and Emerging Opportunities

Haider Saddam Qasim (corresponding author) and Maree Donna Simpson · Tropical Medicine and Infectious Disease, MDPI · 2026

This supplement provides the post hoc classification of the 57 records excluded at title-and-abstract screening and the record of one report sought but not retrieved within the search window. It corresponds to Section 2.7 of the manuscript and to the PRISMA-ScR flow diagram in Figure 1.

Reconstruction note: because screening was conducted by a single reviewer using a written eligibility checklist, the exclusion categories below were reconstructed post hoc from the screening notes. The reconstructed record labels (E01–E57) are internal identifiers used solely to enumerate the excluded set; they do not correspond to unique bibliographic citations and are provided to make the excluded population traceable and auditable.

S1. Summary of Exclusion Categories

Category	Definition	Records (n)
(i)	AI applied in non-travel clinical domains without transferable travel-medicine implications (e.g., imaging, oncology, radiology, mental health, or general primary care AI).	22
(ii)	AI in non-clinical or general-purpose contexts with no travel-medicine relevance (e.g., AI in education, hospitality, tourism marketing, or generic chatbot design).	10
(iii)	Travel behaviour, travel epidemiology or post-travel illness studies without an AI or decision-support component.	12
(iv)	General AI methodology or technical machine-learning / large-language-model papers without a clinical implementation or evaluation component.	7
(v)	Duplicate or near-duplicate coverage of an already-included source, including preprints subsequently superseded by published versions.	4
(vi)	Editorials, opinion, or letters not addressing AI in or near a pre-travel consultation context.	2
Total	Excluded at title-and-abstract screening.	57

S2. Excluded Records — Reconstructed Log (n = 57)

The following records were excluded at title-and-abstract screening. Each row lists the reconstructed record label, a brief content descriptor drawn from the screening notes, the assigned exclusion category, and the exclusion reason.

ID	Content Descriptor (from screening notes)	Exclusion Category	Exclusion Reason
E01	AI-based mammography triage in Australian screening programs.	(i) Non-travel clinical AI	Imaging AI; no transferable pre-travel decision-support implication.
E02	Deep-learning fundus photography for diabetic retinopathy screening.	(i) Non-travel clinical AI	Retinal-image AI; unrelated to travel medicine workflows.
E03	GPT-4 performance on radiology board-style questions.	(i) Non-travel clinical AI	Radiology-only benchmark; findings not transferable to pre-travel counselling.
E04	Machine-learning early-warning score for inpatient sepsis.	(i) Non-travel clinical AI	Inpatient acute-care model; no pre-travel relevance.
E05	AI-driven prostate cancer risk stratification.	(i) Non-travel clinical AI	Oncology decision support; outside PCC concept boundary.
E06	LLM-generated discharge summaries in cardiology.	(i) Non-travel clinical AI	Documentation task in non-travel specialty; no transferable travel implication.
E07	ChatGPT accuracy on USMLE step examinations.	(i) Non-travel clinical AI	Examination performance; not clinical implementation in travel medicine.
E08	AI chatbot for adolescent mental-health screening.	(i) Non-travel clinical AI	Mental-health screening; outside travel-medicine PCC.
E09	ML prediction of ICU readmission using EHR data.	(i) Non-travel clinical AI	Inpatient risk prediction; not pre-travel decision support.
E10	Deep-learning skin-lesion classifier evaluated against dermatologists.	(i) Non-travel clinical AI	Dermatology AI; no travel-medicine transferability.
E11	AI-assisted colonoscopy polyp detection.	(i) Non-travel clinical AI	Endoscopy AI; outside PCC concept.
E12	ChatGPT responses to ophthalmology patient FAQs.	(i) Non-travel clinical AI	Ophthalmology FAQ evaluation; not pre-travel counselling.
E13	LLM performance on paediatric emergency triage vignettes.	(i) Non-travel clinical AI	Emergency triage; not pre-travel prevention.

ID	Content Descriptor (from screening notes)	Exclusion Category	Exclusion Reason
E14	AI-supported antibiotic stewardship recommendations in hospital.	(i) Non-travel clinical AI	Inpatient stewardship; no direct pre-travel counselling analogue.
E15	ML-based sepsis biomarker discovery.	(i) Non-travel clinical AI	Biomarker/omics work; not a clinical implementation study.
E16	ChatGPT evaluated on nephrology questions.	(i) Non-travel clinical AI	Nephrology accuracy benchmark; no travel relevance.
E17	AI risk score for post-operative complications.	(i) Non-travel clinical AI	Perioperative prediction; unrelated to pre-travel counselling.
E18	Chatbot for cancer treatment side-effect self-management.	(i) Non-travel clinical AI	Oncology self-management; outside PCC.
E19	GPT-4 answers to cardiology guideline queries.	(i) Non-travel clinical AI	Cardiology guideline queries; not travel guidance.
E20	AI-based fall-risk assessment in older adults.	(i) Non-travel clinical AI	Fall prevention; not pre-travel counselling.
E21	LLM comparison for orthopaedic patient-education pamphlets.	(i) Non-travel clinical AI	Orthopaedic education; only tangential to travel education.
E22	Machine learning for hospital length-of-stay prediction.	(i) Non-travel clinical AI	Operational prediction; not travel medicine.
E23	AI chatbot for hotel concierge services.	(ii) Non-clinical / general-purpose	Hospitality AI; not clinical.
E24	Generative AI for airline customer-service handling.	(ii) Non-clinical / general-purpose	Non-clinical travel-industry AI.
E25	ChatGPT in higher-education essay marking.	(ii) Non-clinical / general-purpose	Education AI; not clinical.
E26	AI-generated tourism marketing content evaluation.	(ii) Non-clinical / general-purpose	Tourism marketing; no clinical component.
E27	Generic customer-service chatbot design principles.	(ii) Non-clinical / general-purpose	General chatbot UX; not clinical AI.
E28	AI-based travel itinerary optimisation for leisure travellers.	(ii) Non-clinical / general-purpose	Itinerary planning; no health advice component.
E29	LLM-powered language translation quality benchmark.	(ii) Non-clinical / general-purpose	General translation NLP; no clinical implementation.
E30	AI chatbot for banking customer onboarding.	(ii) Non-clinical / general-purpose	Financial services AI; outside PCC.
E31	Generative AI for corporate training modules.	(ii) Non-clinical / general-purpose	Workplace training AI; not clinical.

ID	Content Descriptor (from screening notes)	Exclusion Category	Exclusion Reason
E32	AI in retail voice-assistant satisfaction survey.	(ii) Non-clinical / general-purpose	Consumer-electronics UX; no clinical relevance.
E33	Post-travel malaria case series without AI or decision-support component.	(iii) Travel behaviour/epidemiology, no AI	Post-travel epidemiology; no AI concept.
E34	Cross-sectional survey of VFR traveller knowledge, attitudes, and practices.	(iii) Travel behaviour/epidemiology, no AI	Behavioural survey; no AI/CDSS component.
E35	Traveller uptake of yellow fever vaccine — retrospective clinic audit.	(iii) Travel behaviour/epidemiology, no AI	Clinic audit without AI/CDSS.
E36	Rabies post-exposure prophylaxis after animal contact abroad.	(iii) Travel behaviour/epidemiology, no AI	Post-exposure care; no AI content.
E37	Traveller's diarrhoea aetiology cohort in returning travellers.	(iii) Travel behaviour/epidemiology, no AI	Epidemiological study; no AI component.
E38	Migrant health screening yield in a refugee cohort.	(iii) Travel behaviour/epidemiology, no AI	Migrant health; no AI/CDSS.
E39	Altitude illness incidence among trekkers — observational.	(iii) Travel behaviour/epidemiology, no AI	Epidemiological observation; no AI.
E40	Dengue seroprevalence in expatriates.	(iii) Travel behaviour/epidemiology, no AI	Serology study; no AI/CDSS content.
E41	Traveller vaccination uptake before and after COVID-19 pandemic.	(iii) Travel behaviour/epidemiology, no AI	Uptake analysis; no AI component.
E42	Pre-travel consultation cost analysis in a UK primary-care setting.	(iii) Travel behaviour/epidemiology, no AI	Health-services costing; no AI.
E43	Travel-related sexually transmitted infection risk survey.	(iii) Travel behaviour/epidemiology, no AI	Risk survey; no AI/CDSS component.
E44	Retrospective review of yellow fever vaccine adverse events.	(iii) Travel behaviour/epidemiology, no AI	Vaccine safety analysis; no AI content.
E45	Transformer architecture optimisation for medical text embeddings.	(iv) General AI methodology	Technical NLP; no clinical implementation.

ID	Content Descriptor (from screening notes)	Exclusion Category	Exclusion Reason
E46	Retrieval-augmented generation benchmark on open-domain QA (non-medical).	(iv) General AI methodology	Open-domain NLP; no clinical evaluation.
E47	Fine-tuning strategy for domain-adapted LLMs (theoretical).	(iv) General AI methodology	ML methodology; no clinical or safety component.
E48	Prompt-engineering taxonomy for general-purpose LLMs.	(iv) General AI methodology	Prompt engineering; no clinical implementation.
E49	Chain-of-thought reasoning benchmark on math problems.	(iv) General AI methodology	General reasoning benchmark; no clinical relevance.
E50	Adversarial attacks on general-purpose LLMs.	(iv) General AI methodology	ML security; no clinical implementation context.
E51	Model compression techniques for on-device inference.	(iv) General AI methodology	Systems / engineering paper; no clinical evaluation.
E52	Preprint version of the Singapore Travel Clinic Assistant implementation letter (superseded by peer-reviewed publication [24]).	(v) Duplicate/near-duplicate	Superseded by included version.
E53	Conference-abstract summary of the multi-model hallucination assurance study (superseded by full paper [13]).	(v) Duplicate/near-duplicate	Superseded by included full-text version.
E54	Preprint of the LLM clinical evaluation systematic review (superseded by published version [31]).	(v) Duplicate/near-duplicate	Superseded by included version.
E55	Overlapping editorial by the same author group covering the same content as the included natural-history editorial [10].	(v) Duplicate/near-duplicate	Duplicate coverage of already-included editorial.
E56	Editorial on generative AI hype in medicine (no pre-travel content).	(vi) Editorial / opinion, off-topic	Off-topic opinion; no pre-travel focus.
E57	Letter to the editor on ChatGPT and medical writing ethics.	(vi) Editorial / opinion, off-topic	Ethics of medical writing; not pre-travel decision support.

S3. Retrieval Log — Reports Sought But Not Retrieved

One record proceeded to retrieval but could not be obtained within the search window. No records were excluded after full-text assessment.

ID	Descriptor	Retrieval Steps Attempted	Final Status
R01	A conference proceedings paper (identified via citation chasing) proposing a bespoke pre-travel AI intake prototype at a European travel clinic; full text not accessible through institutional subscription, open-access repositories, or direct author contact within the May 2026 search window.	Sought via publisher website, PubMed link-out, institutional repository, and email request to corresponding author; no response received before the search window closed.	Sought but not retrieved within the search window.

S4. Records Assessed in Full Text and Included

Eleven reports were assessed in full text and all eleven met the eligibility checklist; no reports were excluded after full-text assessment. Full extracted characteristics for these included sources are provided in Supplement S4, and the evidence synthesis with GRADE-informed certainty is presented in Table 3 of the manuscript.
