# Supplementary material for: Artificial Intelligence Tools in Pre-Travel Health Consultations: A Scoping Review of Clinical Evidence, Implementation Gaps, and Emerging Opportunities
Source: Trop Med Infect Dis. 2026 Jul 6;11(7):186. doi: 10.3390/tropicalmed11070186 (PMC13431339; doi:10.3390/tropicalmed11070186)
Supplement: Supplementary file 1 [file tropicalmed-11-00186-s001.zip › Supplement_S4_Characteristics_of_Included_Sources.pdf]

Supplementary Material — Supplement S4

Characteristics of Included Sources — Extended Charting Table

Artificial Intelligence Tools in Pre-Travel Health Consultations: A Scoping Review of Clinical Evidence, Implementation Gaps, and Emerging Opportunities

Haider Saddam Qasim (corresponding author) and Maree Donna Simpson · Tropical Medicine and Infectious Disease, MDPI · 2026

This supplement provides the completed data charting form for the 11 sources included in the scoping review. It corresponds to Sections 2.5 and 3.2–3.3 of the manuscript and expands Table 3 with a fully separated per-field record for each included source. Charting was conducted by a single reviewer using the structured checklist described in Supplement S2.

Source [Ref]	Country / Setting	Source Type	AI / Tool Type	Consultation Task Mapped (CDC Yellow Book) [1]	Sample Size	Outcomes / Comparators	Main Findings	Key Safety Concerns	Implementation Implications	GRADE-Informed Certainty (Reasoning) [43]
Ngiam et al. [23]	Not patient-setting specific (international / online)	Scenario-based expert evaluation of ChatGPT (no patient sample)	General-purpose ChatGPT	General advice, vaccination, malaria prophylaxis, traveller's diarrhoea, vector avoidance	Not applicable (expert scenario review; no patient sample)	Expert benchmarking of ChatGPT outputs against pre-travel advice scenarios	ChatGPT provided readable and often accurate answers to common questions; content quality was uneven for itinerary-specific and comorbidity-adjusted advice.	Generic advice; insufficient itinerary and comorbidity personalisation; no model version or scenario diversity guarantees.	Educational potential only; not a substitute for individualised clinician advice.	Very low (single non-patient study; high indirectness; no comparator)
Koh et al. [24]	Singapore tertiary pre-travel clinic	Implementation research letter with feasibility outcomes	Custom GPT-4 pre-consultation assistant	Pre-consultation education, query elicitation, complex	26 travellers; a small physician group	Traveller and clinician perceived consultation focus	Assistant was acceptable to travellers and physicians; perceived improved	Small sample; self-report outcomes; digital literacy barriers; no EHR	Feasibility signal only, not effectiveness evidence; supports	Very low (small single-site implementation; subjective

Source [Ref]	Country / Setting	Source Type	AI / Tool Type	Consultation Task Mapped (CDC Yellow Book) [1]	Sample Size	Outcomes / Comparators	Main Findings	Key Safety Concerns	Implementation Implications	GRADE-Informed Certainty (Reasoning) [43]
				traveller education		and knowledge benefit; qualitative feedback	consultation focus and knowledge benefit.	integration; hallucination risk.	design of larger evaluation.	outcomes; serious imprecision)
Baglivo et al. [25]	Italy / prototype context	Framework and pre-alpha custom GPT example	Custom GPT prototype (travel-health chatbot)	Personalisation, geolocation, multilingual support, clinic referral, EHR aspiration	Not applicable (framework paper)	Proposal of ten design requirements (decalogue) for safe travel-health chatbots	Proposed ten design requirements — including geolocation, personalisation, multilingual support, clinic referral, and EHR integration — for safe travel-health chatbots.	Prototype lacks full privacy, scope control, and EHR safeguards; no empirical outcomes.	Useful for implementation principles; not an outcome-generating study.	Very low (framework paper; no empirical outcomes)
Flaherty [9]	International travel medicine	Expert opinion / editorial (supervised GenAI integration)	Generative AI broadly	Pre-clinic preparation, translation, literacy tailoring, reminders	Not applicable (editorial)	Not applicable	AI may support preparation and reinforce consultation learning; must remain supervised by clinicians.	Cannot replace individualised clinician judgement; risks amplifying incorrect advice if unsupervised.	Supports supervised, clinician-integrated deployment models.	Very low (expert opinion; non-empirical)

Source [Ref]	Country / Setting	Source Type	AI / Tool Type	Consultation Task Mapped (CDC Yellow Book) [1]	Sample Size	Outcomes / Comparators	Main Findings	Key Safety Concerns	Implementation Implications	GRADE-Informed Certainty (Reasoning) [43]
Flaherty and Piyaphanee [10]	International travel medicine	Expert opinion / editorial (natural-history perspective)	AI broadly	Risk personalisation, behaviour prediction, surveillance	Not applicable (editorial)	Not applicable	Frames AI's potential trajectory in travel medicine including personalisation, behaviour prediction, and surveillance uses.	Non-empirical; aspirational rather than evaluative.	Signals implementation roadmap; not an outcome-generating study.	Very low (expert opinion; non-empirical)
Vibert et al. FeverTravel App [30]	Switzerland; returned-traveller fever workflow	Case–control simulated consultations	Tablet clinical decision-support algorithm	Travel-related risk intake, exposure history, dynamic clinical reasoning	Seven physicians; three simulated patients	Diagnostic reasoning and workflow feasibility in simulated consultations	Demonstrates feasibility issues and adoption considerations for a travel-related CDSS in simulated post-travel consultations.	Indirect to pre-travel prevention; clinician interaction and adoption matter and were not fully solved.	Useful for workflow and adoption lessons transferable to pre-travel CDSS design.	Low (small simulation study; indirect to pre-travel)
CDC Yellow Book [1]	United States guidance (global travellers)	Authoritative clinical guidance	Not AI (reference standard)	Gold-standard task taxonomy for pre-travel risk assessment	Not applicable (guideline)	Not applicable	Defines the pre-travel consultation task taxonomy that AI tools must support and not oversimplify.	Authoritative reference standard against which AI outputs should be checked; risk that AI outputs may	Serves as reference standard for evaluating AI-generated pre-travel advice.	Not applicable (guideline; serves as reference standard)

Source [Ref]	Country / Setting	Source Type	AI / Tool Type	Consultation Task Mapped (CDC Yellow Book) [1]	Sample Size	Outcomes / Comparators	Main Findings	Key Safety Concerns	Implementation Implications	GRADE-Informed Certainty (Reasoning) [43]
								diverge from current guidance.		
ISTM pre-travel advice fact sheet [2]	International travel medicine	Professional fact sheet	Not AI (reference standard)	Risk assessment, timing, vaccines, medicines, chronic illness	Not applicable (guideline)	Not applicable	Reinforces that pre-travel advice extends beyond vaccines and must address timing, medicines, and chronic illness.	Useful patient-facing standard; AI must not understate timing-of-consultation criticality.	Serves as scope reminder for AI-generated pre-travel content.	Not applicable (guideline; serves as reference standard)
WHO International Travel and Health — Malaria [3]	Global guidance	Authoritative clinical guidance	Not AI (reference standard)	Malaria geography, chemoprophylaxis, mosquito protection	Not applicable (guideline)	Not applicable	Defines a high-risk domain requiring up-to-date recommendations that change frequently.	Updates frequently; AI tools relying on training-data snapshots may be outdated.	Highlights the recency requirement for AI advice in malaria chemoprophylaxis and prevention.	Not applicable (guideline; serves as reference standard)
Collins multi-model hallucination assurance [13]	General clinical decision support	Simulation study; multiple LLMs	Multiple LLMs	Safety testing for clinical decision support (including transferability to pre-travel	Multiple models across multiple prompt sets	Fabrication rate and elaboration of false clinical details across models	LLMs repeated or elaborated false clinical details in 50–82 percent of outputs across	Directly relevant to hallucination risk in AI-generated travel advice, including vaccine, dose, and	Supports conservative deployment models with clinician-in-the-loop oversight.	Moderate for general LLM hallucination risk; indirect for travel (consistent

Source [Ref]	Country / Setting	Source Type	AI / Tool Type	Consultation Task Mapped (CDC Yellow Book) [1]	Sample Size	Outcomes / Comparators	Main Findings	Key Safety Concerns	Implementation Implications	GRADE-Informed Certainty (Reasoning) [43]
				guidance content)			evaluated models.	prophylaxis content.		multi-model finding)
Asgari et al. CREOLA hallucination framework [14]	Clinical documentation	Framework and evaluation study	LLMs (documentation task)	Clinical documentation accuracy; extrapolated to AI-generated travel advice	Documentation samples across evaluators	Prevalence of hallucination versus omission, by document section	Hallucinations were more often “major” than omissions, especially in plan sections.	Directly relevant to AI-generated travel plans (recommendations, prophylaxis regimens) where hallucinated content carries clinical risk.	Supports minimum hallucination-reporting standards for pre-travel AI evaluations.	Low to moderate (single framework study; high indirectness to travel)

Legend

• GRADE-informed certainty is applied conceptually (per Section 2.6 of the manuscript). Editorials and guideline texts are appraised using JBI text-and-opinion guidance and are not assigned a formal GRADE rating.

• Consultation task mapping follows the CDC Yellow Book pre-travel consultation taxonomy [Ref 1].

• Reference numbers refer to the reference list in the main manuscript (49 references total).
