# Supplementary material for: Artificial Intelligence Tools in Pre-Travel Health Consultations: A Scoping Review of Clinical Evidence, Implementation Gaps, and Emerging Opportunities
Source: Trop Med Infect Dis. 2026 Jul 6;11(7):186. doi: 10.3390/tropicalmed11070186 (PMC13431339; doi:10.3390/tropicalmed11070186)
Supplement: Supplementary file 1 [file tropicalmed-11-00186-s001.zip › Supplement_S5_Reporting_Standards_and_Tool_Inventory.pdf]

Supplementary Material — Supplement S5

Minimum Reporting Standards Checklist and Tool Inventory

Artificial Intelligence Tools in Pre-Travel Health Consultations: A Scoping Review of Clinical Evidence, Implementation Gaps, and Emerging Opportunities

Haider Saddam Qasim (corresponding author) and Maree Donna Simpson · Tropical Medicine and Infectious Disease, MDPI · 2026

This supplement records (a) the minimum reporting standards that future pre-travel AI evaluations should meet — mapped item-by-item to CONSORT-AI, SPIRIT-AI, TRIPOD+AI, and DECIDE-AI — and (b) an inventory of commercial and openly accessible AI tools currently used or proposed for pre-travel health consultations, together with their current evidence limitations. It corresponds to the discussion of implementation gaps and reporting standards in Sections 4 and 5 of the manuscript.

S1. CONSORT-AI Checklist — Applicability to Pre-Travel AI Trials

CONSORT-AI extends the CONSORT reporting standard to randomised controlled trials of AI interventions. It applies prospectively to any future travel-medicine AI RCT; the current scoping evidence base does not yet include such trials. The items below are those most directly relevant to pre-travel AI evaluation.

CONSORT-AI Item	Description	Applicability to Pre-Travel AI Evidence Base
CONSORT-AI 1a/1b	Title and abstract identification of AI intervention	Applies to future RCTs of pre-travel AI tools. This scoping review is not an RCT; the item is flagged as a requirement for confirmatory trials rather than as a review item.
CONSORT-AI 4a(i)	Inclusion and exclusion criteria at participant and input-data level	Future travel-medicine AI RCTs should specify eligibility both for participants and for the input data the AI consumes (e.g., traveller history fields).
CONSORT-AI 5(i)	Detailed description of the AI intervention including version	None of the four direct travel-medicine AI sources reports the AI version with sufficient granularity for reproduction; flagged as an evidence gap.
CONSORT-AI 5(iv)	Instructions and skills required for delivery	Not reported in the four included direct travel-medicine AI sources.
CONSORT-AI 5(v)	Setting where the AI intervention was integrated	Partially reported by Koh et al. (Singapore tertiary travel clinic); not reported by other direct sources.
CONSORT-AI 5(vi)	Human–AI interaction and handover procedures	Not systematically reported; flagged as a priority evidence gap for supervised deployment.
CONSORT-AI 6a(i)	Analysis of the performance error of the AI	Not reported in the included empirical direct source (n = 26); flagged as a priority for future trials.
CONSORT-AI 19	Generalisability to other contexts and populations	Only single-site feasibility evidence exists; multi-site, multi-population generalisability remains unstudied.

CONSORT-AI Item	Description	Applicability to Pre-Travel AI Evidence Base
CONSORT-AI 22	Data sharing and code availability	Not reported in the included direct sources; flagged as a reproducibility gap for future confirmatory work.

S2. SPIRIT-AI Checklist — Minimum Reporting for Future Pre-Travel AI Protocols

SPIRIT-AI extends the SPIRIT protocol reporting standard to AI-intervention trials. It applies to protocols; any future pre-travel AI RCT should register a SPIRIT-AI-compliant protocol.

SPIRIT-AI Item	Description	Recommended Minimum for Pre-Travel AI Protocols
SPIRIT-AI 6a	Rationale for AI use in the trial	For any future pre-travel AI RCT, the rationale should link to the specific pre-travel consultation task (per CDC Yellow Book).
SPIRIT-AI 11a(i)	Description of AI intervention including model architecture, training data, and version	A minimum reporting requirement for any subsequent pre-travel AI protocol. Not yet met in the current evidence base.
SPIRIT-AI 11a(iv)	Human–AI interaction procedures	Recommended minimum: a documented handover pathway from AI output to supervising clinician, with escalation triggers.
SPIRIT-AI 12	Outcomes including safety outcomes	Recommended minimum: hallucination rate, guideline-fabrication rate, and escalation-appropriateness rate as prespecified safety outcomes.
SPIRIT-AI 20a	Statistical methods including subgroup analyses for equity-relevant strata	Recommended minimum: pre-specified analyses across language, VFR status, and digital-literacy strata.

S3. TRIPOD+AI Checklist — Minimum Reporting for Pre-Travel AI Prediction Models

TRIPOD+AI updates the TRIPOD standard for prediction model studies that use AI. It applies to any future travel-medicine AI risk-prediction study.

TRIPOD+AI Item	Description	Recommended Minimum for Pre-Travel Prediction Model Studies
TRIPOD+AI 1	Identification as an AI-based prediction model study	For any future travel-medicine AI risk-prediction model, the study should be identified as such in the title and abstract.
TRIPOD+AI 5	Data source and eligibility	A minimum reporting requirement: source of traveller data used to train and evaluate any pre-travel risk model.
TRIPOD+AI 8	Predictors including features derived from the AI	Recommended minimum: full disclosure of features and any derived embeddings used in a travel-risk model.

TRIPOD+AI Item	Description	Recommended Minimum for Pre-Travel Prediction Model Studies
TRIPOD+AI 11	Model specification, hyperparameters, and version	Recommended minimum: reproducible model card with version pinning.
TRIPOD+AI 17	Performance measures including calibration and equity metrics	Recommended minimum: calibration in-the-large, subgroup calibration, and net benefit analysis.
TRIPOD+AI 20	Limitations including fairness and generalisability	Recommended minimum: explicit statement of population, geography, and time-window limits.

S4. DECIDE-AI Checklist — Minimum Reporting for Early-Stage Pre-Travel AI Evaluation

DECIDE-AI applies to early-stage live clinical evaluation of AI decision-support systems and is directly relevant to the Koh et al. Singapore feasibility study [Ref 24] and to any subsequent pre-travel AI feasibility work.

DECIDE-AI Item	Description	Applicability to Pre-Travel AI Feasibility Studies
DECIDE-AI 1	Reporting of the early-stage clinical evaluation of an AI decision support system	Applies to Koh et al. Singapore feasibility study [24] and to future pre-travel AI feasibility studies.
DECIDE-AI 4	Human factors including workflow integration	A minimum requirement for any future travel-medicine AI feasibility report.
DECIDE-AI 6	Learning curve of clinician users	Not yet reported for pre-travel AI tools; flagged as an evidence gap.
DECIDE-AI 12	Safety-related events including hallucinations and clinically significant errors	Recommended minimum for any pre-travel AI feasibility study.
DECIDE-AI 15	Modifications to the AI during evaluation	Recommended minimum: change-log for any prompt, tool, or model change during evaluation.

S5. Inventory of Commercial and Enterprise AI Tools Relevant to Pre-Travel Care

The following inventory lists categories of commercial and enterprise AI tools currently used or proposed for pre-travel workflows, together with their present evidence limitations. Reference numbers refer to the manuscript reference list (49 references total).

Tool Category	Description	Deployment Setting	Current Evidence Limitations	Recommended Use Pending Confirmatory Evidence
General-purpose consumer LLM chatbots (e.g., ChatGPT free and paid tiers, Google Gemini consumer, Anthropic Claude consumer,	General-purpose LLM assistants used ad hoc by travellers and clinicians for pre-travel questions.	Public web and mobile applications.	Not designed or regulated as clinical decision-support systems; not aligned with CDC Yellow Book, WHO International Travel and Health, or ISTM guidance; hallucination and fabrication of authoritative guideline	Should not be used by clinicians as a source of pre-travel decisions; may be used by travellers only with clear caveats and

Tool Category	Description	Deployment Setting	Current Evidence Limitations	Recommended Use Pending Confirmatory Evidence
Microsoft Copilot consumer)			content documented across multiple models (see Refs [13,32]).	clinician follow-up.
Enterprise health-focused LLM assistants (e.g., health-vertical wrappers on general LLMs)	Enterprise deployments of general-purpose LLMs with health-specific system prompts.	Enterprise health systems and consumer health apps.	Reduce but do not eliminate hallucination risk; travel-medicine-specific safety evidence is not yet established.	Deploy only with clinician-in-the-loop supervision, hallucination monitoring, and hard scope controls.
Custom GPT / bespoke prototype pre-travel assistants (e.g., Koh et al. Singapore assistant [24]; Baglivo et al. decalogue prototype [25])	Custom GPT-4 or equivalent assistants built for a specific pre-travel clinic workflow.	Single-site or research-context deployments.	Only one implementation study (n = 26) with subjective feasibility outcomes; no comparative effectiveness or safety evidence; no EHR integration; hallucination risk not systematically measured.	Suitable only for supervised feasibility evaluation and prototype refinement; not ready for routine clinical deployment.
Tablet clinical decision-support systems for travel-related workflows (e.g., Vibert et al. FeverTravelApp [30])	Rule-based or hybrid clinical decision-support systems for travel-related clinical reasoning.	Research prototypes in tertiary travel or infectious-diseases services.	Simulated feasibility evidence only; not evaluated in true pre-travel counselling; workflow adoption challenges reported.	Useful for design lessons on clinician–CDSS interaction; not ready for pre-travel deployment.
Retrieval-augmented generation (RAG) medical assistants (e.g., configurations described in [38])	LLMs constrained to authoritative source documents via retrieval augmentation.	Research and enterprise prototypes.	Improved accuracy and generalisability across languages in general medical QA; no travel-medicine-specific evaluation identified.	Recommended architectural direction for future pre-travel AI, provided sources are pinned to CDC Yellow Book, WHO, and ISTM guidance and updated on their release cycle.

S6. Inventory of Openly Accessible AI Tools and Resources

This inventory covers openly accessible AI tools and reference resources relevant to pre-travel care and to future evaluation of pre-travel AI systems.

Tool / Resource Category	Description	Current Travel-Medicine Evidence	Alignment with Authoritative Guidance	Recommended Use
Openly accessible generic chatbots and consumer LLMs used for pre-travel queries	Free-tier ChatGPT, Gemini, Claude, Copilot, and open-web chatbots.	Extensive general-medicine literature but no rigorous travel-medicine evaluation beyond scenario studies [23]; documented hallucination and guideline fabrication across models [13,32].	Absent — not designed to comply with CDC, WHO, or ISTM guidance and not maintained against guideline update cycles.	Not recommended as a standalone source of pre-travel decisions; may be used by travellers only with explicit clinician follow-up.
Openly accessible travel-focused non-clinical chatbots (e.g., itinerary planners, travel-industry customer service bots)	General-purpose travel and hospitality chatbots.	None identified within the review scope that address clinical pre-travel counselling.	Absent.	Out of clinical scope; should not be presented to travellers as a source of pre-travel health advice.
Openly accessible clinical AI safety and reporting-standards resources (e.g., CONSORT-AI, SPIRIT-AI, TRIPOD+AI, DECIDE-AI documentation)	Reporting-standards checklists and explanatory documents.	Directly applicable to future travel-medicine AI evaluations.	Not a clinical tool per se; provides the reporting minimum.	Recommended as the reporting baseline for any future pre-travel AI evaluation.
Openly accessible authoritative travel-medicine guidance (CDC Yellow Book, WHO International Travel and Health, ISTM fact sheets)	Reference-standard clinical guidance.	Not AI tools; serve as the reference standard against which AI-generated pre-travel advice should be checked.	Not applicable.	Recommended as the authoritative source pinned into any future pre-travel AI system (e.g., via retrieval augmentation).

S7. Summary of Minimum Reporting Recommendations for Future Pre-Travel AI Evidence

- Prospectively register any pre-travel AI RCT protocol against SPIRIT-AI, and report the completed trial against CONSORT-AI.
- Report the AI model, version, training-data window, prompt architecture, and human–AI handover procedure with sufficient granularity for reproduction.
- Pre-specify hallucination rate, guideline-fabrication rate, and escalation-appropriateness rate as safety outcomes, and report them by traveller subgroup (language, VFR status, digital-literacy stratum).
- Pin authoritative travel-medicine guidance (CDC Yellow Book, WHO International Travel and Health, ISTM fact sheets) into the AI system via retrieval augmentation with a documented update cycle.
- Report equity and fairness analyses across pre-specified strata, including VFR travellers, older adults, First Nations Australian and Pacific Islander communities, and limited-English-proficiency travellers.
- Publish the model card, prompt library, and evaluation dataset schema alongside the primary report, consistent with TRIPOD+AI data-sharing recommendations.
